# Rhizomal Reclassification of Living Organisms

**DOI:** 10.3390/ijms22115643

**Published:** 2021-05-26

**Authors:** Ahmad Ibrahim, Philippe Colson, Vicky Merhej, Rita Zgheib, Mohamad Maatouk, Sabrina Naud, Fadi Bittar, Didier Raoult

**Affiliations:** 1 IHU Méditerranée Infection, 13005 Marseille, France; ahmad.ibrahim@etu.univ-amu.fr (A.I.); philippe.COLSON@univ-amu.fr (P.C.); vicky.merhej@univ-amu.fr (V.M.); rita.zgheib@etu.univ-amu.fr (R.Z.); mohamad.maatouk@etu.univ-amu.fr (M.M.); sabrina.naud@etu.univ-amu.fr (S.N.); 2 Aix-Marseille Université, IRD, APHM, MEPHI, 13005 Marseille, France; 3 Aix-Marseille Université, IRD, APHM, SSA, VITROME, 13005 Marseille, France

**Keywords:** evolution, tree of life, sequences transfer, reclassification, lifestyle, rhizome, TRUC, candidate phyla radiation, giant virus, Asgard

## Abstract

Living organisms interact with each other during their lifetime, leading to genomes rearrangement and sequences transfer. These well-known phenomena give these organisms mosaic genomes, which challenge their classification. Moreover, many findings occurred between the IXXth and XXIst century, especially the discovery of giant viruses and candidate phyla radiation (CPR). Here, we tried to provide an updated classification, which integrates 216 representative genomes of the current described organisms. The reclassification was expressed through a genetic network based on the total genomic content, not on a single gene to represent the tree of life. This rhizomal exploration represents, more accurately, the evolutionary relationships among the studied species. Our analyses show a separated branch named fifth TRUC (Things Resisting Uncompleted Classifications). This taxon groups CPRs together, independently from Bacteria, Archaea (which regrouped also Nanoarchaeota and Asgard members), Eukarya, and the giant viruses (recognized recently as fourth TRUC). Finally, the broadening of analysis methods will lead to the discovery of new organisms, which justify the importance of updating the classification at every opportunity. In this perspective, our pragmatic representation could be adjusted along with the progress of evolutionary studies.

## 1. Introduction

**Figure 1:**
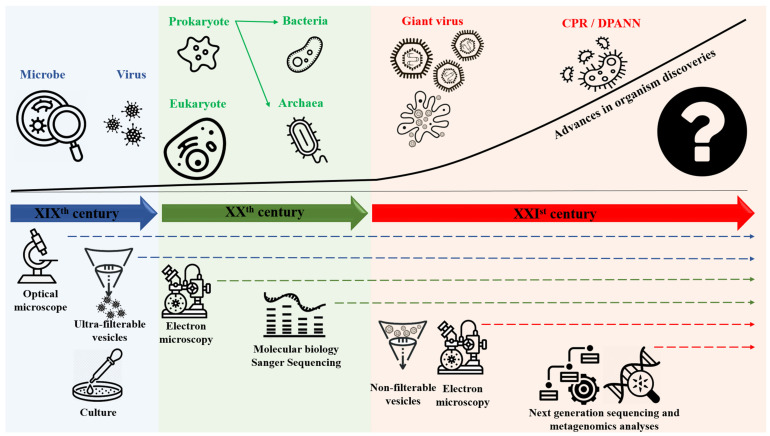
Global timeline of organism discovering and classification depending on the technologies used.

Devising a broadly accepted classification system for microorganisms and living things, in general, depends on the tools that are available at a given time to enable the definition of these organisms (Table 1) [1]. During the 19th century, optical microscopy made it possible to define organisms that were only visible with this instrument as microbes (Table 1) according to L. Pasteur [2]. Following the first application of the culture method by R. Koch in 1882 [3], these microorganisms have been differentiated into two groups according to Gram staining in 1884 [4]. At the end of the 19th century and for several decades thereafter, increasing evidence emerged of entities that caused infectious diseases but were not microbes: They were invisible by optical microscopy, ultra-filterable using a Chamberland filter, and did not multiply on inert culture media; these entities were gradually characterized with the advent of cellular cultures and were eventually named viruses (Table 1) [5,6,7,8] (Figure 1). During the 20th century, progressive inventions significantly increased our knowledge of the microbial world. In 1925, using light microscopy; E. Chatton classified microbes based on their structure: prokaryotic cells were devoid of a nucleus, while eukaryotic cells contained a nucleus [9]. Moreover, electron microscopy was invented in 1935, and the first micrograph of a biological specimen was produced in 1937 [10,11]. This invention allowed us to observe and characterize microorganisms/objects/corpuscles not described before [10,11]. Then, in the second half of the 20th century, the advent of molecular biology led the world of microbes to be divided into three branches (Archaea, Bacteria, and Eukarya) (Table 1) (Figure 1), named domains by C. Woese, largely based on the analysis of small subunits of ribosomes namely 16S ribosomal RNA in prokaryotes and 18S ribosomal RNA in eukaryotes [12]. Nonetheless, some criteria of established classifications have been challenged over time. Thus, bacteria in the phylum *Planctomycetes* (such as *Gemmata obscuriglobus*) were determined to have membranes surrounding their nucleoids [13,14]. Additionally, eukaryotic cells were observed to contain remnants of symbiotic (Table 1) bacteria (*Alphaproteobacteria*) in the form of mitochondria (Table 1) and the border between strict intracellular bacteria (Table 1) and mitochondria appeared to be unclear [15]. The 21st century has made the picture still more complex, as notable improvements have been made in technologies and analysis tools. Cultures on amoebae combined with electron microscopy observations enabled the discovery of a new generation of viruses: Giant viruses (Table 1) that have particle sizes, genome sizes, and complexities comparable to those of small putative microbes [16,17]. Still, more recently, metagenomics revealed intermediates between archaea and eukaryotic microorganisms named Asgard (*Asgardarchaeota*) (Table 1) [18]. Finally, metagenomics unveiled two groups of very small (<300 nm) microbes. The first ones are classified in the Candidate Phyla Radiation (CPR) bacteria (Table 1) [19,20] that includes the TM7-*Saccharibacteria* phylum [21] and seem to be bacterial exosymbionts. We named them the “mini-microbes.” The other ones are Nanoarchaea (Table 1) (or DPANN for *Diapherotrites*, *Parvarchaeota*, *Aenigmarchaeota*, *Nanoarchaeota,* and *Nanohaloarchaeota*), a group of very small archaea that depend on symbiotic interactions with other archaea [22,23] (Table 1, Figure 1).

These microorganisms multiply and interact with each other within ecological niches where they encounter or that are the fields of their obligatory relationship, which allows transfers of genetic sequences between their genomes [24]. This generates new species harboring mosaic genomes, i.e., composed of sequences of various origins and of unknown sequences [25]. It is, therefore, necessary to analyze the whole genome sequence of these organisms to decipher their evolutionary history comprehensively and classify them accurately [26]. In phylogenetics, rooted trees are based on one or several genomic fragments that can have distinct evolutionary histories but show a single history [26,27,28]. In contrast, rhizomes (Table 1) are more appropriate representations of the pattern pluralism of genetic evolution on a genome-scale than trees are [29,30]. Indeed, rhizomes consider unprogressive descent as a result of lateral sequence transfers (Table 1) in addition to vertical inheritance [26,27,28]. This paradigm contradicts the theory, which suggests that all organisms originate from a single ancestor (currently named the Last Universal Common Ancestor (LUCA)) (Table 1) and that each node in the tree of Life (Table 1) represents the common ancestor of related organisms [31,32]. Due to this evolutionary complexity, and as it appears to be futile to define whether microorganisms are living entities or have a parasitic lifestyle (Table 1), it is necessary at present to propose a new classification of living microorganisms. This classification is based on both phenotypic and genotypic characteristics, including the lifestyle of these microorganisms; markers of genome evolution, including the presence/absence of informational genes, translation components, and protein folds; and the phylogeny of very conserved genes. In this work, we studied the evolutionary history of representative microorganisms and the mosaicism (Table 1) of their genomes. In addition, we have considered for the first time all living microorganisms larger than 300 nanometers and harboring both DNA and RNA. We attempted to gather all these data in an effort to redefine and reclassify these microorganisms in the 21st century as objects containing autonomous genetic information that live alone or in association with other organisms and are identified as a unique corpuscle.

## 2. Genome Evolution and Mosaicism Representation: Rhizomes Construction

In the 21st century, identifying a universal common ancestor for all living organisms is no longer realistic [2,33,34]. From a taxonomic perspective, living organisms have been classified following the use of ribosomal RNA genes as markers of identity. This classification has divided the tree of life into three primary domains, Eukaryota, Archaea, and Bacteria [12,35,36], which does not include microorganisms without ribosomes, such as giant viruses, or newly discovered phyla, such as CPR and DPANN Recently, Banfields’ group reconstructed the tree of life by including CPR and DPANN [19]. In spite of the high diversity within CPR lineages, these microbes were clustered together into a distinct branch of the tree and subdivided the bacterial domain [19,20]. Moreover, taxonomic characterization based on a single gene means that there is a unique origin for each organism, which excludes and overlooks evidence of the extensive transfer of sequences among living organisms [31] and genome reduction process due to a specific niche adaptation [25,30,37]. This oversight also limits the evolution of the prokaryotes, which makes the data insufficient [38]. A rhizome might be a better representation of genetic evolution. This model considers the mosaicism of genomes and that their sequences can result from exchanges, fusions, recombination, recruitment, degradation, or de novo creation. Therefore, de novo creation, chimeric genes, and ORFans (Table 1) are considered [39]. Sequence transfer performed between living organisms in life may provide an explanation for the high genetic diversity observed at the genome level [30,39,40,41]. A phylogenetic tree is based on the analysis of one gene or a group of genes. Thus, a traditional tree of life cannot represent an entire genome. The origin of living organisms and their evolutionary history is better represented by a rhizome, which considers the whole genome sequence [26].

**Figure 2:**
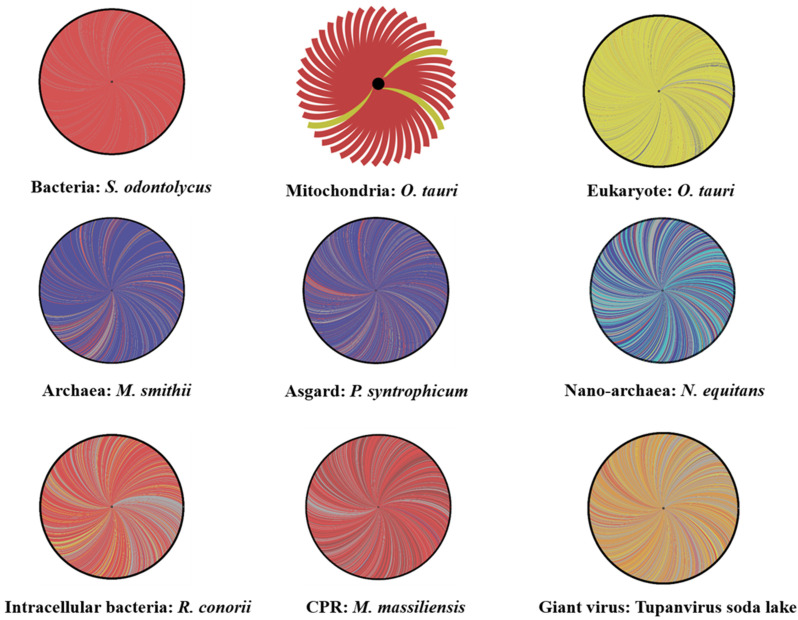
Rhizomes of genomes illustrative of the mosaicism of each life domain including: *Schaalia odontolycus* (a bacterium), *Rickettsia conorii* (an intracellular bacteria), *Minimicrobia massiliensis* (a CPR), Tupanvirus soda lake (a Mimivirus), *Nanoarchaeum equitans* (a nano-archaea), *Methanobrevibacter smithii* (an archaea), *Prometheoarchaeum syntrophicum* (an Asgard), *Ostreococcus tauri* (a eukaryote) and its mitochondria. Each gene is represented by a curve, coloring according to the origin: bacterial origin in dark red, CPR origin in purple, Eukarya origin in yellow, virus origin in orange, archaea origin in dark blue, nano-archaea origin in light blue, and orfans in grey. The figures were performed using the Gephi online tool.

The genome from a member of each cellular domain or group studied in this research, preferentially microorganisms isolated in our institute was selected for rhizome construction. These members included a eukaryote, *Ostreococcus tauri* (the smallest free-living eukaryote with organelles (mitochondria and chloroplast) [42]), and its mitochondrion and chloroplast; an Asgard, *Prometheoarchaeum syntrophicum*; an archaeon, *Methanobrevibacter smithii*; a nanoarchaea, *Nanoarchaeum equitans*; an intracellular bacteria, *Rickettsia conorii*; a classical bacterium, *Actinomyces odontolycus* (recently reclassified and renamed *Schaalia odontolyticus*); a CPR, *Minimicrobia massiliensis* (the first *Saccharibacteria sp.* whose genome was assembled from a stool sample in our research laboratory—CADDWL00000000); and a giant virus, Tupanvirus soda lake (Figure 2). BLASTp searches were performed for each gene product against the NCBI protein sequence database. After exclusion from the best matches of the sequences of organisms belonging to the same genus or taxon as query sequences, the remaining best hits were selected based on several criteria: amino acid identity >20%, sequence coverage >30%, and e-value <0.001 [43]. Next, rhizomes of genomes were constructed using Gephi software (https://gephi.org/, accessed on 23 May 2021). The set of selected HITs for each genome was divided according to taxonomic categories of the NCBI database. The rhizomes, in a single figure for all genes from a given genome, show the taxonomy of the best BLASTp hits that are putative donors or acceptors involved in sequence transfers, as well as the ORFans (sequences devoid of homologs in databases). Our objective was to evaluate and compare the mosaicism of all organisms. We attributed the origin of all studied sequences following their taxonomic membership in NCBI.

Then, in order to compare the intra-group evolutionary profile, an additional rhizome has been added for each domain/division. This analysis includes *Reclinomonas americana* for eukaryote, and its mitochondrion, *Lokiarchaeum* spp., for Asgard, *Ignicoccus hospitalis* for archaea, *Nanopusillus acidilobi* for DPANN, *Treponema pallidum* for bacteria, *Candidatus uzinura diaspidicola* for intracellular bacteria, *Candidatus Gracilibacteria bacterium* HOT-871 for CPR, Cedratvirus massiliensis for giant virus and the chloroplast of *Micromonas commoda.*

### 2.1. Rhizome of All Archaeal Members

For Asgard, the representative member (*P. syntrophicum*), which was analyzed, showed that 76% of its genes were linked with Archaea, 11% of the genes were linked with bacteria (430 genes), and 0.38% of the genes were linked with eukaryotes (15 genes out of 3944). Only 11% of the genes were unique to the genome of *P. syntrophicum* (ORFans concerning the genome). Then, for comparison, we also studied the mosaicism of an archaeon (*M. smithii*) and a eukaryote *(O. tauri*). A total of 82% of the encoding genes for *M. smithii* originated from archaea, 9% from bacteria, and 0.5% from eukaryotic genes (Figure 2).

We noticed that the mosaic profiles of this archaea and Asgard were proportionate. Both profiles share a close genomic repertoire that is different from that of eukaryotes (86% eukaryotic genes, 2% archaeal genes, 2.4% bacterial genes, and 9% ORFan genes). This common genomic repertoire, composed primarily of genes linked to archaeal and bacterial members, confirms the grouping of Asgard in the same clade as archaea in hierarchical clustering and phylogeny. Even the DPANN members (*N. equitans*) share a similar gene repertoire with *M. smithii* and *P. syntrophicum* (42% of genes with archaea, 29% with other nano archaea genes, 4% from bacteria, 1% eukaryotic sequences, and 22% ORFans). Notably, the high percentage of sequences matched with bacteria (compared to other domains) is explained by the high sequence exchange between archaea and bacteria that host the same ecological niche [24,44]. This finding suggests that these organisms have the same evolutionary history and that they evolved together independently of eukaryotes. Furthermore, recent studies have shown the presence of many eukaryotic signature proteins in the genomes of some Asgard members, which may be due to the horizontal sequence transfer described above [45].

**Figure 3:**
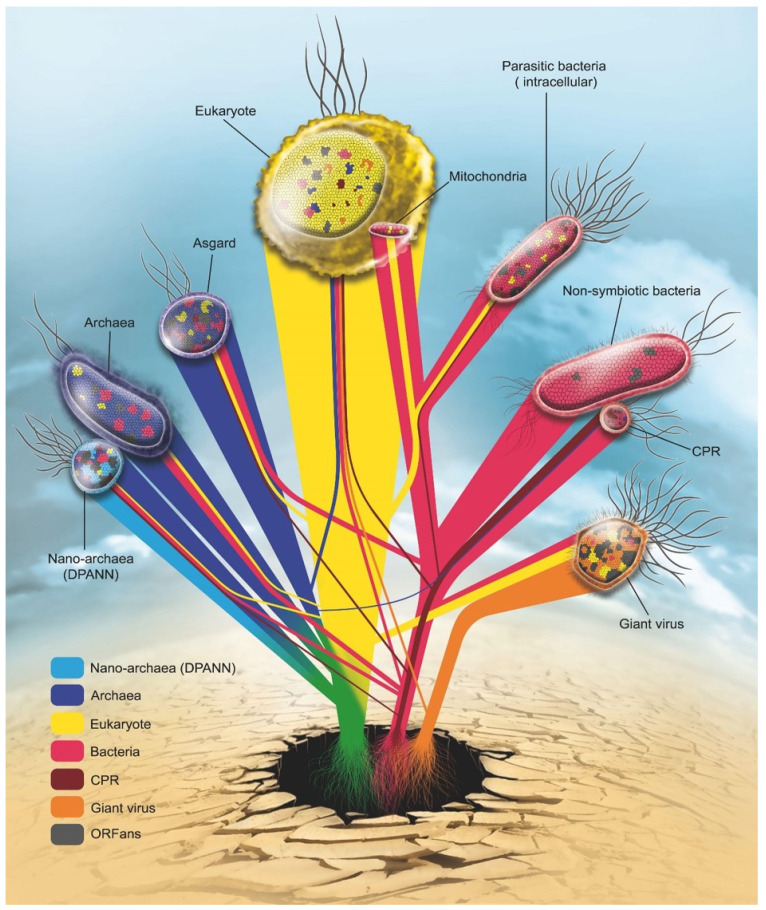
Rhizome of life: Roots of each gene are represented according to the current reclassification of living organisms: Eukarya (yellow), Archaea (blue), Nano archaea (light blue), Bacteria (red), CPR (dark red) Giant virus (orange). In grey are genes without identified origin (ORFans).

The idea of sequence transfers between Asgard and eukaryotes was confirmed following the analysis of eukaryotic signature proteins [46]. These genes have different architectures than classical eukaryotes, as well as differences in their function. These differences in dynamic evolution could be originated from horizontal gene transfer, gene duplication, gene loss [46]. In addition, the distribution of these genes is not equal among all Asgard species, which is logically explained by the loss of genes and the transfer of sequences [47]. Hence, the common evolutionary background of eukaryotes and archaea and, more precisely, the Asgard can be represented by a deeper unknown common ancestor [46,47,48]. This ancestor (marked in green in Figure 3) presents outside the current classification and the eukaryotic and archaeal diversity currently described. The ancestor may present components of a eukaryote (the essential set of eukaryotic genes) [48,49], which may subsequently be accumulated or transferred within and between Asgard and eukaryotes [45,46].

Even though Asgard species have eukaryotic proteins in their genomes, their mosaic profile and genetic variability led us to classify them with archaea. This classification is due to the rhizome, which is not based on a gene or set of genes that may be transferred. In this study, according to our analyses, we confirmed that Asgard and DPANN represent two separated archaeal phyla.

### 2.2. Rhizome of Eukaryote

For eukaryotes, the origin is probably the most controversial. Eukaryotes were the first example of the massive transfer of sequences with the bacterial symbiosis from which mitochondria originate [15]. In a number of cases, mitochondrial sequences have migrated within the chromosomes of eukaryotes without leaving traces [50]. In other cases, mitochondria are present, as in the louse, as a set of individual plasmids that recombine [15]. The association of proto-eukaryotes with bacteria of the *Alphaproteobacteria* group is a phenomenon that is generally accepted [51]. This association means that if our estimate of the age of *Alphaproteobacteria* is on the order of 1 billion years, eukaryotes, as we know them, are the result of a bottleneck that occurred approximately a billion years ago. In addition, plants have also benefited from a second bacterial symbiont, cyanobacteria, from which the chloroplast is derived. It was integrated within the plant/algue’s cells and contributed additional genes/functions to their host (Photosynthesis). [42,52]. In fact, from the observation of mitochondria, we knew that a monophony of living organisms was impossible. However, for a long time, this phenomenon was considered to be an exception and not the rule, which was only belatedly challenged by Doolittle and Bapteste [53].

The origin of proto-eukaryotes is also the subject of robust debate. It appears that trees made with the three original putative domains show a clearer association of proto-eukaryotes with archaea than with bacteria, and many of the signatures currently observed in eukaryotes seem to originate from archaea [54,55]. This finding led to the belief that eukaryotes had partially evolved from archaea. Another hypothesis emerged, which proposed that the nucleus of eukaryotes, which differentiates them from prokaryotes, originated from giant viruses, considering that the most conserved genes in giant viruses appear to have a deep branching in eukaryotes, although it is unclear whether the source of these genes is eukaryotic or prokaryotic [56].

Finally, the extent of genetic exchanges between eukaryotes and other organisms they encountered has been recently elucidated with the number and importance of integrated retroviruses in the genomes of various hosts, including humans (more than 10,000 in this case) [57]. In addition, the ease with which very long sequences can integrate into the genome of eukaryotes from that of their respective parasites has also contributed to these exchanges. Thus, *Wolbachia* sp. is able to integrate up to 80% of its chromosome into the DNA of its hosts (Spiders, Insects, *Wuchereria bancrofti*) [58]. For example, genomic analyses of the genome of *Drosophila ananassae* showed the presence of the genome of *Wolbachia* sp., which has obligatory intracellular activity [59]. Additionally, in humans, the human herpesvirus type 6 virus (HHV-6) is entirely capable of integrating into the genome of humans and of being transferred from parents to children [60], as are sequences of *Trypanosoma cruzi* [61]. Moreover, some studies showed that eukaryotic cells are also exposed to bacterial/archaeal DNA transfer [62,63]. Thus, these are transformed through the natural ability of some bacteria to integrate their DNA into the genome of their host (mostly eukaryotic). Bacteria are surrounded by eukaryotes in the same ecological context. Recent reports have indicated the presence of bacterial DNA fragments for *Bartonella henselae*, *Rhizobium etli*, *Escherichia coli*, etc., in eukaryotic genomes. [62,63]. Eukaryotes, therefore, show considerable complexity relative to their origins, which are multiple. We cannot clearly define what proto-eukaryotes arise from. Current eukaryotes appeared relatively recently, and they exhibit mosaicism that is not simply a reflection of past encounters, but that is still developing at present. The example of the endogenization of retroviruses in koalas in the 20th century shows that the mosaicism phenomenon remains underway [57,64]. In the other hand, virus endogenization is not limited to retrovirus; it also concerns giant viruses [65,66]. The representation of eukaryotic mosaicism shows a high prevalence of Endogenous Viral Elements (EVEs) [65]. Some double-stranded DNA viruses can also intrude into the eukaryotic genome. For example, numerous nucleotide signatures of NCLDV (giant virus) have been detected in some green algae’s genomes [65,67]. It is known that these corpuscles are highly mosaic, with genes of multiple origins. These integrations provide new genetic materials in eukaryotic cells that affect the composition of their own genomes in the future [65,66,67]. Moreover, several studies suggest that eukaryotic nuclei are originated from an ancient NCLDV [56]. Finally, with the recent demonstration of the integration of many giant virus genes inside amoebae [68,69], the mosaicism of eukaryotes appears to involve several bacteria, archaea, and other organisms [70]. The genome of a eukaryote (amoeba, plants, etc.) may contain sequences from hosted microorganisms [71], such as giant viruses, bacteria/cyanobacteria, and candidate phyla radiation [72]. Similarly, virophages in their algal hosts [67,73,74] show that this phenomenon is the rule and not the exception, as had been hypothesized during the discovery of mitochondria. The mosaicism of the eukaryotic genome is also confirmed in this study at the level of *O. tauri* genomes, which contain some genes from the bacterial and archaeal domains (Figure 2). The presence of chimeric genes is always possible, regardless of the domain studied, which makes the rhizome a highly reliable model in determining the history of evolution that is closest to reality (Figure 3).

### 2.3. Giant Viruses: Rhizome of Living Viruses

Viruses have long been neglected by evolutionists. Viruses are considered to be the most abundant corpuscles on the planet, especially in the environment. Following metagenomics analyses, genes of viral origin have the highest percentage of the genosphere [14]. Traditional viruses may be defined as obligate intracellular parasites that infect the three main life domains (archaea, eukaryotes, and bacteria (infected by bacteriophages)). Viruses can infect other viruses (virophage: a parasite that parasitizes a parasite); on the other hand, they are characterized by their absence of ribosomal genes, as well as the presence of only one type of nucleic acid (either RNA or DNA), very small genomes and total independence at the level of replication. According to Lwoff, viruses are not living organisms (unlike the 3 domains of life); he suggests that in all pathogenic organisms, the infectious agent is the organism itself, except for viruses, where the infectious agent is represented by the nucleic acid alone. These characteristics make viruses unique corpuscles that are not comparable to classical domains.

On the other hand, the discovery of giant viruses in 2003 [16] changed the general representation of the classification. Giant viruses are viruses with a very large physical size (larger than some bacteria); they have a large genome compared to classical viruses (up to 1 to 2 megabases), as well as both types of nucleic acids. In addition, a recent study has shown that some giant viruses also have enzymes capable of producing energy (with the detection of the tricarboxylic acid cycle and proton gradient in Pandoravirus massiliensis) [75]. For this reason, only giant viruses were analyzed in this work. Specifically, we chose Tupanvirus soda lake as a representative because it has the largest translation component set of all the giant viruses examined in the study.

**Figure 4:**
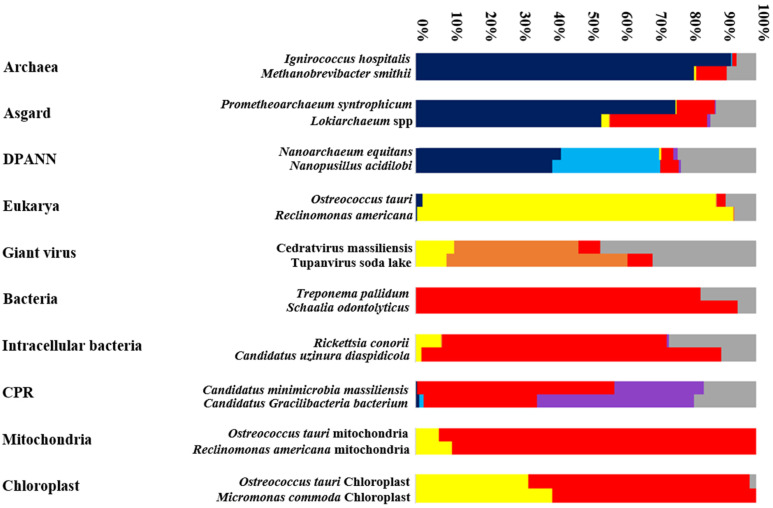
A graphical representation showing the distribution (percentage) of genes in a given genome according to their origin: Archaea (Dark blue), DPANN (Light blue), Eukarya (Yellow), Bacteria (Red), CPR (Purple), Giant virus (Orange) and ORFans (Grey).

The analysis of the protein sequences of Tupanvirus soda lake demonstrates strong mosaicism (Figure 2 and Figure 4). The genetic repertoire of these sequences consists of 53% of a mixture of viral sequences, 9% with eukaryotes, and 7% with bacteria. Interestingly, it is important to note that more than 30% of the sequences of this genome are unique (387 ORFans/1276) (Figure 2 and Figure 4). This diverse origin is probably due to a strong exchange of sequences between viruses and other microorganisms. Giant viruses infects eukaryotic cells, such as algae or amoebae [76,77]. This lifestyle leads to the coexistence of several parasites and/or symbionts in the same amoeba. Subsequently, the giant virus is exposed to a group of multisource genes [69,78]. This type of interaction likely produces organisms with highly mosaic and chimeric genomes, such as giant viruses [43,78]. An important component of the viral genome consists of eukaryotic and bacterial proteins, as demonstrated by the rhizome of Tupanvirus soda lake.

Thus, organisms with reduced genomes show an abundance of ORFans (30.33%, 25.61%, 22.8%, and 15.19% for Tupanvirus soda lake, *N. equitans*, *R. conorii*, and *M. massiliensis*, respectively) (Figure 2 and Figure 4). Some of these ORFan sequences are probably newly created through fusion, gene degradation, deletion, or sequence transfer [26,79]. Thus, we can assume that the more abundant the ORFan sequences are, the greater the mosaicism of the genome of an organism is. This group of sequences was not analyzed by a phylogenetic tree. Thus, to predict the origin of an organism, we strongly emphasize the importance of analyzing the mosaic composition of an organism’s given genome (Figure 3) [25].

### 2.4. Do CPR and Bacterial Species Have the Same Mosaic Profile?

In addition, we compared candidate phyla radiation (CPR) mosaicism and bacteria. We found that the genetic repertoire of *S. odontolyticus* (classical bacteria) is the most homogeneous genome, consisting only of bacterial sequences (95%) and sequences unique to this species (5%). On the other hand, a non-negligible percentage of the R. conorii genome is composed of eukaryotic proteins (7.66%) (Figure 2 and Figure 4).

Regardless of the bacteria’s lifestyle, either intracellular or not, communication between microorganisms leads to genetic exchange between them [21]. Therefore, intracellular bacteria (*R. conorii*) tend to exhibit more mosaic genomes due to their interactions with the eukaryotic host and their interactions with other bacteria that colonize the same host [80,81]. In this study, we showed that the genetic variability at the genome level of *R. conorii* is higher than that of *S. odontolyticus*. Lateral/horizontal sequence transfer in bacteria is well-known, since bacteria present mobile elements, plasmids, and transposons in their genomes that facilitate genetic exchange [82]. This transfer can also be mediated by bacteriophage infections [83,84], or vesicles DNA transportation to its bacterial host [85]. Hence, the homogeneity at the genome level of *S. odontolyticus* described in this study is not representative, and the genomic analysis of other bacteria shows a high rate of mosaicism. For example, *Neisseria gonorrhoeae* is a strictly human pathogenic bacterium; therefore, it interacts with human cells through a pathogenic parasitic relationship. Analysis of the *N. gonorrhoeae* genome shows the presence of eukaryotic sequences (human nuclear elements) [86]. In addition, it has been shown that a high percentage (25%) of the *Thermotoga maritima* genome is linked with archaea [39]. This bacterium is considered a thermophilic environmental microbe that shares an ecological niche with archaea. Therefore, this transfer is also possible between two fields that share an environment, even though neither one affects the other.

The exchange of sequences participates in bacterial diversity and evolution [83]. Genome evolution is also possible through the phenomenon of sequence loss, which leads to organisms with reduced genomes, as in the case of *Rickettsia* [38]. This variability at the level of the bacterial genome, achieved through daily communication, is more accurately modeled by a rhizome representative of the whole genome.

Moreover, the CPR genome presents particular mosaicism, where for *Minimicrobia massiliensis*, 506 genes have bacterial sequences (54.12%), 282 CPR genes (30.16%) have sequences from other CPR phyla (*Parcubacteria*, *Microgenomates*, and unclassified *Patescibacteria*), 142 genes are ORFans (15.19%), and 5 genes have archaeal sequences (0.53%) (Figure 2). This mosaic pattern shows that the transfer profile of the sequences is established in general only with bacteria. This finding suggests that CPR has symbiotic (or parasitic) activity only with bacteria and not any other domain (which is not the case with giant viruses, for example). CPR also exhibited a large portion of their own protein sequences (30.16 ± 15.19 = 45.35%).

In addition, CPR species have notably reduced genomes (measuring approximately 1 Mb) [19]. Consequently, it is possible that these species have undergone the phenomenon of sequence loss to evolve. CPR genomes are composed of a high percentage of hypothetical proteins (more than 40% in some genomes); therefore, most of their biosynthetic and metabolic activities remain unknown to date.

In addition, the introns present in CPR genomes at the level of their ribosomal genes and their transfer RNA [87] give rise to the special characteristics of CPR species that lead them to be classified in a single division (Figure 3), as they present a lifestyle, evolutionary history and genome diversity that are unique and different from the species in the bacterial domain.

### 2.5. Organelles’ Rhizomes: Mitochondria and Chloroplast

In this study, we analyzed the organelles of the green algue: *O. tauri*, the smallest eukaryote that contains both organelles: mitochondria and chloroplast. As we predicted, most of the mitochondrial genes of *O. tauri* are of bacterial origin (93%), while 7% of the mitochondrial genes are derived from eukaryotic sequences (Figure 2). It has been shown that the mosaicism of a mitochondrial genome is a result of transfer from different sources, mostly bacterial (specifically the class of *Alphaproteobacteria* but not exclusively the order of *Rickettsia*). Moreover, 25% of *O. tauri* mitochondria genes belong to the *Gammaproteobacteria* class, which confirms that the creation of mitochondrial genes is a result of several successive events and is not limited to a single type of bacteria [15,25,38]. Mitochondria interact with eukaryotic cells through durable symbiotic interactions. A mitochondrial genome differs from one species to another: for example, a single-chromosomal genome for the *Reclinomonas americana* and *Saccharomyces cerevisiae* mitochondria [15] versus a multichromosomal genome for the *Pediculus humanus* mitochondria (18 minichromosomes) [88]. The events of mitochondrial creation are not stable; therefore, their evolution is also different. It has also been shown that there is a different ancestor for a given mitochondrion [89]. This theory has been confirmed by examining the diversity obtained in our analyses of the mitochondria of *O. tauri*.

Concerning *O. tauri*’s chloroplast, our analyses confirm, similarly to mitochondria, its major bacterial origin [42]. This origin is not limited only to *Terrabacteria* group (cyanobacteria), some genes with proteobacterial origin were found (≈48%). We also show a potential high occurrence of sequence transfer from its eukaryotic host (an average of 34% eukaryotic genes founded in *O. tauri*’s chloroplast genome) [42,90,91] (Figure 4). This is another example of endosymbiotic lifestyle [91], showing both bacterial and eukaryotic mosaicisms due to the gene loss and sequence transfer carried out between chloroplast and its eukaryotic host [42,91].

These results support the notion that mitochondrial and chloroplast mosaicism should also be presented by a rhizome, as one tree is not sufficient to explain the chimeric state of these genomes (Figure 3).

### 2.6. Genetic Network: The Representation of Interactions between Microbes

It is impossible to have a last unique common ancestor for all organisms, especially since organisms have large differences in their sequences [92,93]. The life domains thus share most of the sequences exchanged among them, which has been shown in the constructed genetic network (Figure 3). This network shows the communication between the studied species and the evolutionary history of each species. It is clear that eukaryotes have followed their own path of evolution, independent of archaeal groups (Archaea, Nanoarchaeota, and Asgard), which is the case for CPR species that evolved with bacteria at the same time and not from bacteria. The CPR species have a unique evolutionary path that is independent of bacteria. Mitochondria/Chloroplast evolved from bacteria and was hosted by eukaryotes following a symbiotic association while retaining only the essential genes. This interaction gives an additive transfer from the host.

Evolutionary history is increasingly complex, and the construction of a corresponding all-inclusive tree of life that details the classification of living organisms is even more complicated. Following the intra-group evolutionary profile comparison, each group of corpuscles shows common mosaicism (Figure 4). A rhizome of two different species belonging to the same group shows more or less the same evolutionary history. This may be due to their stable lifestyle or the common ecological niche they inhabit. For example, all CPR/DPANN are exo-symbiotic to bacteria/archaea, respectively, and all mitochondria/Chloroplast are endo-symbiotic to eukaryotic cells. The mosaic profile of an organisms’ group always depends on their environment (or neighboring microbes) [65]. A transfer of genetic information between them is always possible, so each gene presents a unique story. Again, this complexity shows that the whole genome analyses give a better explanation of an organism’s evolution (Figure 4). The taxonomic characterization of an organism, or a group of organisms, cannot be based on a single sequence or group of sequences (sequence concatenation) [31]. It has already been shown that phylogeny is disrupted by a significant percentage of chimeric sequences in each domain [25]. In addition, a microbial species can have different variants of 16S rRNA [94], and determining which of these copies should be the basis of phylogeny is impossible. This problem is another argument against classifying organisms based on the 16S ribosomal RNA phylogeny alone. Thus, evolution, as we see in Figure 3, is a gathered set of roots, with each representing an origin of a group of genes, and some of them are still largely unknown [31,38]. The construction of a genetic rhizome is a more reliable and logical means of representing the origin of species than is a tree-based on limited data. Therefore, studies of the evolution of organisms should evolve, as well.

## 3. Hierarchical Clustering Based on Informational COGs and Fold Superfamily Domains: A Symbiotic Lifestyle Branch along with the Three Domains of Life

Hierarchical clustering analysis was performed based on the presence/absence patterns of the orthologs of 737 informational COGs in the genomes from 216 representatives of the three domains of life (Eukarya, Archaea, and Bacteria), nanoarchaea (DPANN, for *Diapherotrites*, *Parvarchaeota*, *Aenigmarchaeota*, *Nanoarchaeota*, and *Nanohaloarchaeota*), candidate phyla radiation (CPR; those with a complete genome available on NCBI up to the 1st of December 2019), Asgard (*Asgardarchaeota*) members, various mitochondria (from *Acanthamoeba castellanii*, *Anopheles gambia, Pediculus humanus*, *Homo sapiens*, *Reclinomonas americana, and Saccharomyces cerevisiae*) and giant viruses. The sets of nucleic and amino acid sequences from these species were downloaded from the NCBI (National Center for Biotechnology Information) GenBank non redundant sequence database. Accession numbers of all the sequences used in this study are listed in Table S1 (Supplementary Data). For non-annotated NCBI genomes, genome annotation was performed using Prokka [95].

To detect the genes involved in information storage and processing (so-named “informational genes”) in these genomes, sequence comparisons were performed between their whole repertoire of genes and sequences representative from all clusters of orthologous groups of proteins (COGs) related to informational storage and processing and to nucleotide transport and metabolism (737 COGs) [J, A, K, L, B, and F categories] using Diamond [96] and Proteinortho [97] programs, utilizing as thresholds a minimal identity of 30% and a maximal e-value of 0.001 [43]. Next, we performed a hierarchical clustering analysis based on the presence/absence patterns of genes encoding proteins assigned to each informational COG after eliminating all protein sequences that did not correspond to the aforementioned informational COGs. Thus, the character present or absent for such genes were used to construct a matrix by assigning a “0” if there was no ortholog in the genome and “1” if there was at least one ortholog for a given informational COG. The matrix of Euclidean distance was calculated from this 0/1 matrix, and a dendrogram tree was subsequently constructed by hierarchical clustering with MeV (MultiExperiment Viewer) software (https://www.mev.tm4.org/, accessed on 23 May 2021). Additionally, hierarchical clustering analyses were performed with the same methodology and the same matrix format (0/1) but using only orthologs of COGs in the translation category (J). Moreover, a third hierarchical clustering analysis was performed based on a set of 606 protein fold sequences (fold superfamily domains (FSFs)) downloaded from the SCOP database—version 1.75 (https://www.scop.berkeley.edu/, accessed on 23 May 2021) [98]. Cladograms were displayed using MEGA X software (https://www.megasoftware.net/, accessed on 23 May 2021).

**Figure 5:**
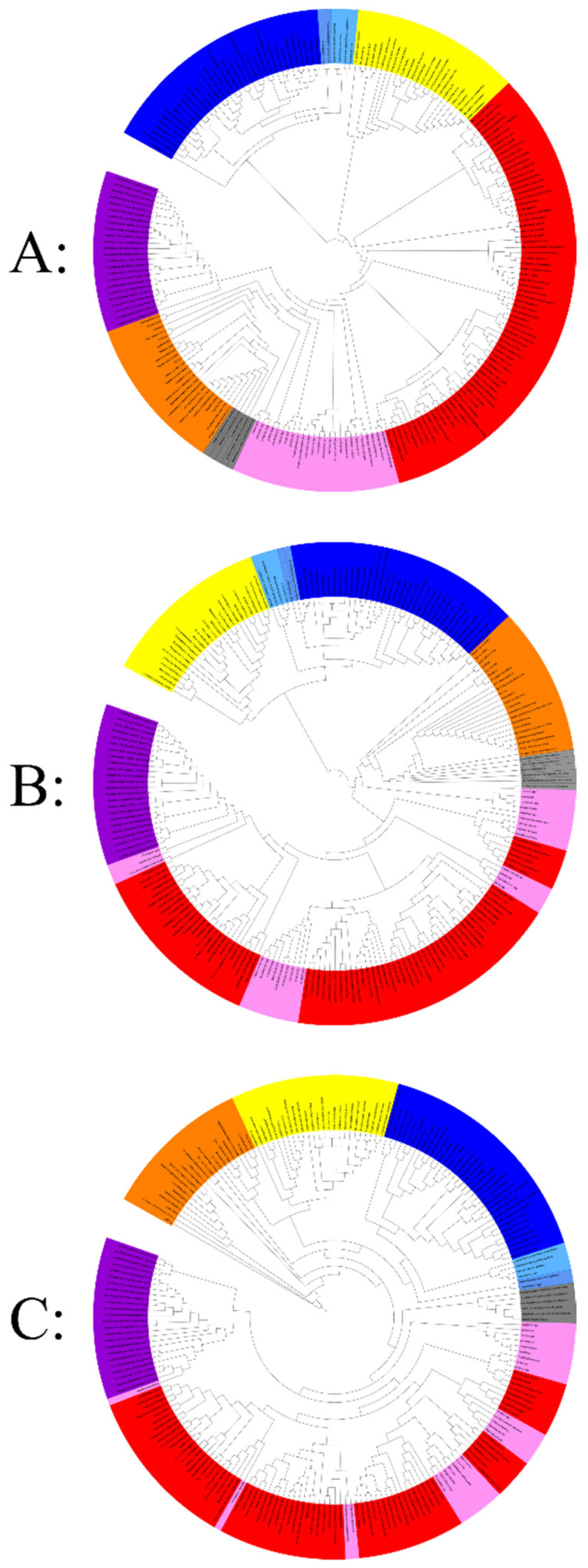
Hierarchical clustering by phyletic pattern based on the presence/absence of (**A**) 737 informational Clusters of Orthologous Groups (COGs) of proteins, (**B**) 180 translational Clusters of Orthologous Groups (COGs) of proteins, (**C**) 606 fold proteins superfamily. The Eukarya members are represented in yellow, Archaea, Asgard, and nano-archaea members in blue (dark to light respectively), classical bacteria members in red, intracellular bacteria members in pink, giant virus members in orange, mitochondria members in grey, and CPR members in purple. Each clustering was provided separately in Supplementary Data 1.

The informational COG clustering showed the topology of a tree with five separate clades (Figure 5A). The first clade regroups the archaea, nanoarchaea, and the two used members of *Asgardarchaeota* who are grouped with the archaea in the same branch, independent of the eukaryotes. This shows that the Asgard have the same set of functional genes as the archaea and that they exhibit the same lifestyle. The second clade corresponds to all eukaryote members; they present a unique and distinctive set of COGs that is incomparable to the other life domains selected in these analyses.

In the same way, all bacteria are clustered together in the same branch. We noticed the presence of two subgroups: the first subgroup corresponded to all classical bacteria, and the second subgroup represented all intracellular bacteria. Then, the mitochondrial genomes and giant viruses regrouped together in a fourth branch, given their strong absence of informational COGs compared to the other members examined; it is important to note that among the mitochondria, *Reclinomonas americana* clustered with intracellular bacteria at the limit with the fourth cluster (which represents giant viruses and mitochondria) because of their particular genome, which is larger than the other mitochondria used in this study: 69,034 bp with the presence of 8 informational COGs (its genome size is the highest of all mitochondria tested genomes). Finally, the fifth branch represents the candidate phyla radiation, which has clustered independently of the bacterial branch (classical and intracellular). These latter species are characterized by their limited set of informational COGs compared to members of the three other branches (Figure 5). Thus, our clustering grouped these organisms and mitochondria depending on their lifestyles.

Concerning the hierarchical clustering analysis based on the translation-related COGs (Category-J) (Figure 5B), the topology of the tree shows that eukaryotes are always independent, as well as archaea (always associated with DPANN and Asgard). In addition, this clustering shows that all the classical bacteria used in this study present the same profile at the level of translational genes. On the other hand, no well-organized taxa representing intracellular bacteria have been observed, and they are dispersed with classical bacteria and giant viruses grouping together. Ultimately, the cluster representing the CPR is always maintained in this analysis, and they always cluster together since they present unique ribosomal structures and sequences different from those of bacteria [87]. It is important to note that the absence of informational COGs does not depend on genome size, the nanoarchaea have very small genomes, and they have a set of COGs comparable to those of archaea and Asgard (they are always clustered in the same branch). Similar clustering was also noticed for classical and intracellular bacteria.

Furthermore, the third clustering based on the protein fold super families (FSFs) shows a different topology (Figure 5C). As described above, intracellular bacteria are not organized in single taxa; they are distributed among the different clades of the tree. According to the fold analysis, All CPRs are represented in a unique subclade of the taxa described above. Only the eukaryote clade was always maintained in all 3 clusters. CPR are characterized by their reduced genomes and inability to synthesize nucleotides de novo [99]. They only manufacture proteins essential for their symbiotic lifestyle, such as pili that are involved in cell-cell interactions [100]. Some proteins present in bacteria are absent in the majority of CPR members [99], such as the CRISPR viral defense leading to a natural resistance to bacteriophages [101]. They also possess a set of 106 protein families that are absent or less abundant in classical/intracellular bacteria [99]. This simplicity at the level of their genomes gives them unique characteristics that cannot be compared to those of bacteria. The use of different methods of hierarchical clustering that are based on the existence of these protein families have grouped CPR differently [99]. This new ramification in the tree of life is independent of other bacteria, even those with symbiotic activity, regardless of the data used. Accordingly, it is suggested that CPR has been co-evolved with bacteria, based on the simplicity of their genomes [99].

## 4. Phylogenetic Analysis of Ancestral Coding and Noncoding rRNA Genes

A BLASTp search was performed for the collected amino acid sequence set mentioned above against a previously described set of conserved genes encoding the DNA-dependent RNA polymerase subunit II (*RNAP II*), DNA polymerase A, ribonucleotide reductase (*RNR*), topoisomerase IIA (*TopoIIA*) (also known as gyrase in bacteria and CPR), elongation factor 1 (*EF-1*), thymidylate synthase (*ThyA*), flap endonuclease 1 (*FEN1*) and proliferating cell nuclear antigen (*PCNA*) sequences (*PCNA* and *FEN1* are observed only in eukaryotes, archaea, nanoarchaea, Asgard and some giant viruses) [78]. Protein sequences found as best HITs were selected using the following thresholds: a minimum alignment length of 70 amino acids, a maximum e-value of 0.001, and a minimum percentage of identity of 20%. In addition, nucleotide sequences corresponding to the 16S rRNA gene (18S rRNA for eukaryotes) and 23S rRNA gene (28S rRNA for eukaryotes) genes were downloaded from the NCBI GenBank nucleotide sequence database [78].

A multiple alignment of protein or nucleotide sequences was performed using MUSCLE software [102]. All sequences were collected following BLAST analyses of the published sequences of these genes [78] against the genomes examined in this study. Next, curated alignments were used for phylogenetic reconstruction with the maximum likelihood (ML) method using nearest-neighbor-interchange (NNI) with the Jones–Taylor–Thornton (JTT) model for the protein sequences and the Kimura 2 substitution model for the nucleotide sequences. Trees were constructed using the MEGA X software

**Figure 6:**
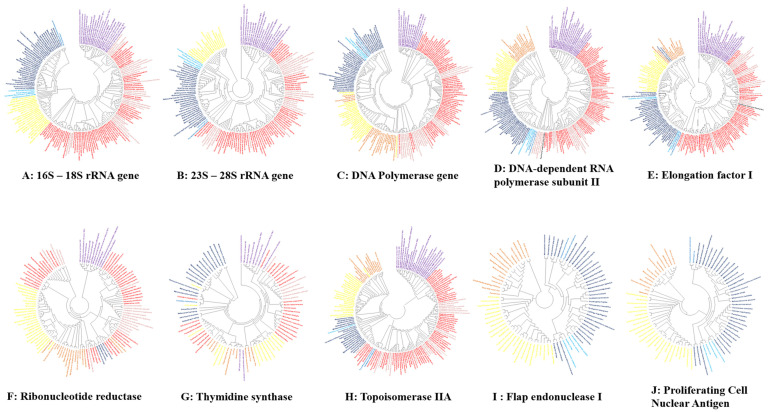
Unrooted phylogenetic trees show the analyses of ribosomal genes and the most conserved genes of giant viruses and the other domains. The eukarya members are represented in yellow, archaea members in dark blue, nano archaea members in light-blue, Asgard members in blue, classical bacteria members in red, intracellular bacteria members in pink, giant virus members in yellow mitochondria in black and CPR members in purple. Each tree was provided separately in Supplementary Data 2.

All phylogenetic trees were built based on the alignment of the retrieved protein sequences of bacteria, CPR, archaea, DPANN, Asgard, eukaryotes, and giant viruses (Figure 6). Regarding the phylogenetic trees based on noncoding ribosomal genes, the analysis of 16S RNA (18S for eukaryotes) and 23S RNA (28S for eukaryotes) for all genomes used in this study (except giant viruses and mitochondria) clearly shows the same topology (similar to that of the COG cladogram), and the clades are always maintained. In particular, intracellular bacteria are completely distributed between the classical bacteria in the same branch without any interference in the taxa representing the CPR (Figure 6). This cluster is grouped separately. On the other hand, this phylogeny shows that the ribosomal sequences of the Asgard members are closer to those of the archaea than to the eukaryotes, and they are also regrouped with the archaea in the same taxa, independent of eukaryotic members (Figure 6).

Concerning the other analyzed ancestral genes, in the six constructed trees, the branches representing CPR and archaea were always maintained, Asgard and DPANN members were always grouped with classical archaea, and no other species interfered with clustering. Intracellular bacteria have never been grouped independently from classical bacteria. The giant viruses always overlap the branch of the eukaryote. In addition, as previously described [78], the two genes *FEN-1* and *PCNA* were not detected in the bacteria or in the tested CPR. However, following our analysis, the RNR gene was determined to be absent in all members of the archaea, Asgard and DPANN tested. In addition, of all the mitochondria tested, only that of *Reclinomonas americana* had the following genes: *RNAP* and *EF-1* (Figure 6).

Following the genetic and protein analysis of the tested organisms, including all microbes larger than 200 nanometers that we gathered together for the first time in this study, and in terms of the origin of the most conserved genes, we can observe that there is one branch containing the bacteria, while another one contains all the archaea, nanoarchaea and Asgard that seem to constitute a group that evolved mostly from a common root, while the CPR species remain a distinct “monophyletic group” and seem to have a single origin that is different from that of the bacteria.

In addition, the intracellular bacteria and the CPR clustered at a distance from the nonsymbiotic bacteria. Notably, in the past, intracellular bacteria were considered intermediate organisms between bacteria and viruses based on their phenotypes [103], which is reflected by their phenotypic traits and parasitic lifestyle. Globally, we believe that there are different levels of organism clustering; autonomous bacteria constitute a well-defined group, both phylogenetically and phenotypically. Similarly, the CPR species constitute, genetically and phenotypically, a single group. Intracellular bacteria constitute, phenotypically, a separate group but do not genetically; this also is the case of mitochondria (symbiotic alphaproteobacteria hosting by some eukaryotic cells). The giant viruses represent phenotypically and genetically homogeneous and heterogeneous groups, respectively.

This heterogeneity may be attributable to the reorganization of genomes by sequence transfer (especially with eukaryotes), as has been demonstrated for Tupanvirus soda lake, as some of its genes are mosaic genes [43]. Furthermore, the archaeal group, which includes the nanoarchaea and Asgard that are never characterized as being intermediate between eukaryotes and archaea [104] in this work but always as being one of the branches of the archaea, constitutes a unique (monophyletic) taxon with divergences that could have occurred within this group following its individualization.

## 5. Conclusions

The phylogenies of the ancestral genes that were constructed in this study, as well as the hierarchical clustering based on the three different sets of proteins and the unique characteristics of CPR [87,99], show a new division in the tree of life: candidate phyla radiation (CPR). The exceptional characteristics of giant viruses cause them to be considered a fourth TRUC of microbes [2,43,105,106]. In this study, analyses of the genomes updated in the database show an additional branch (fifth TRUC) emerging from the same tree next to the giant viruses [43]. This group represents candidate phyla radiation, which cannot be considered classical bacteria or bacterial phyla. These species present particular mosaicism, unique 16S ribosomal RNA genes [20], and an exceptional lifestyle never described previously in classical bacteria [87]. Finally, our results, which are based on all the protein repertoires of these organisms, support the new shape of the tree of life, generated by Banfield’s team in 2016 based on ribosomal proteins [19,20]. At the same time, this study supports that Asgard is an archaean phylum [45] and that the ancestor of eukaryotes remains unknown. Our analyses showed that eukaryotes always cluster outside the archaean branch, and this latter branch always includes the two studied Asgard genomes. On the other hand, microbe interactions occurring over billions of years have resulted in genetic exchanges among them [107], which causes a given genome to exhibit mosaic structure. Therefore, the updated classification of living organisms is represented in this study by a genetic network. This classification is based on the total genomic content and not on a gene or group of genes that cannot be representative of a total genome.

In general, as evolutionary studies should also evolve, our representation of this classification cannot be considered final. With the broadening of the analysis methods, new branches or domains may be discovered, which makes it necessary to update the classification of living organisms at every opportunity.

To the best of our knowledge, this report describes the first attempt to synthesize, in the 21st century, all the data concerning living organisms and to redefine groups of these organisms in the sense of objects containing autonomous genetic information, living alone or in association with other living organisms, and being identified as corpuscles.

## Figures and Tables

**Table 1:** Definition of principle terms used in this analytical review.

Term	Definition
Archaeon	Formerly known as archaebacteria, archaea are unicellular microorganisms measuring between 2 and 15 µm that were initially detected in extreme environments. Archaea have been classified in a separate domain from bacteria and eukaryotes. These organisms have transcription and translation machineries like those of eukaryotes.
Asgard	This superphylum consists of anaerobic prokaryotic microorganisms classified in the domain Archaea. The members of this superphylum are assumed to have an ancestor that is intermediate to archaea and eukaryotes. These organisms are considered to be most genetically similar to eukaryotes with a genome harboring some signature eukaryotic genes.
Bacterium	These unicellular, anucleate (prokaryotic) microorganisms have mainly a single chromosomal genome, different cell shapes and a physical size of between 1 and 10 µm. However, very large bacteria, attaining 750 µm in diameter, and very small bacteria (300 nm) have been described. Some of these organisms may host circular DNA fragments, known as plasmids. Bacteria do not have energetic organelles (mitochondria and chloroplasts); but they produce ATP by generating a proton gradient across their cell membranes through glycolysis. However, some bacteria (cyanobacteria) can use light to generate a proton gradient. Bacteria are divided into different groups according to their cell membrane morphology/Structure: Gram-positive bacteria (also known as monoderm bacteria) have a cell membrane primarily composed of peptidoglycan that retains crystal violet dye after Gram staining; Gram-negative bacteria (so called diderm bacteria) do not retain crystal violet dye after Gram staining because they have a thinner peptidoglycan layer surrounded by an outer membrane containing lipopolysaccharide; Gram-variable bacteria stain irregularly according to the peptidoglycan quantity during cell growth and Gram-indeterminate bacteria, such as *Mycobacterium* spp., which have a waxy layer on its surface preventing them to respond well to Gram staining. Finally, some bacteria lack a cell membrane, like *Mycoplasma* spp.
Candidate (Candidatus)	The term “candidate” refers to an undescribed species or to a single isolate of unknown species for which there is insufficient information for it to be identified as a new species according to the International Code of Bacterial Nomenclature. This term can be assigned also to uncultured prokaryotic organisms obtained by metagenomic analyses.
Candidate Phyla Radiation (CPR)	These ultra-small microbes have a physical size between 100 and 300 nm and a small genome (<1 megabase pair). CPR members are unable to multiply on their own and are dependent on an exo-symbiotic or parasitic interaction with bacteria. These organisms were first detected by metagenomics analyses. CPR members have a unique, characteristic 16S ribosomal RNA gene.
Commensalism	Eating at the same table: This term implies a biological interaction between two organisms without any advantage or disadvantage.
Eukaryotes	These unicellular or pluri-cellular (micro) organism have a nucleus surrounded by a membrane. The size of individual eukaryotic cells is between 10 and 50 µm. These organisms are characterized by their genomes, which are composed of several chromosomes, and some eukaryotes possess mitochondria and/or chloroplasts (plant cells) and other organelles (e.g., endoplasmic reticulum and Golgi apparatus). Eukaryote is a major branch in the tree of life alongside bacteria and archaea.
Genome mosaicism	This term refers to the presence in a genome of sequences with different evolutionary histories, including some that may be currently unknown.
Giant virus	These viruses, formerly known as nucleocytoplasmic large DNA viruses, belong to the phylum *Nucleocytoviricota* and the order Megavirales. The viruses have a larger physical size (>200 nm, non-filterable using Chamberland filter, visible using a light microscope) and genome size (>300,000 base pairs) in comparison to classical viruses; their size is comparable to those of microbes. The giant-virus virion contains both DNA and RNA, which includes messenger RNAs and transfer RNA, and the genome often harbors genes encoding translation components and a specific mobilome, an energy production system in some cases, which are absent in classical viruses’ genomes. Giant viruses comprise a monophyletic group with an ancient origin that may predate that of eukaryotes, and these viruses have a broad host range.
Intracellular bacteria	These bacteria (also known as obligate intracellular parasites) are unable to multiply independently and require host eukaryotic cells to develop and reproduce, like *Rickettsia* spp. These microbes are considered facultative intracellular bacteria, if they have the ability to grow inside and outside eukaryotic cells independently like *Bartonella henselae* and *Listeria monocytogenes*.
Lateral sequence transfer	This term refers to the transfer of genetic information (regardless of whether sequences include full-length ORFs or partial ORFs) between the genomes of a donor species and a recipient species, regardless of their evolutionary relationship.
LUCA (Last Universal Common Ancestor)	This organism is considered to be the starting point of life on earth. A virtual and controversial entity that would represent the common ancestor of all (micro) organisms.
Microbe—Microorganism	These organisms are visible by microscopy (due to a size >200 nm).
Mitochondria	These eukaryotic organelles possess the characteristics of prokaryotic cells, ranging in size from 0.5 to 1 µm. Mitochondria are present in most eukaryotic cells and produce ATP, providing energy for the cell. Genetically, mitochondria are of alpha-proteobacterial origin.
Chloroplast	Eukaryotic organelle of plant and algal cells with a size ranging from 1 to 2 µm conducts photosynthesis. Genetically, Chloroplast are of Cyanobacteria origin.
Mutualism	This term implies a lasting association between two dependent organisms with mutual benefit and no negative effect on either organism. Both organisms can reproduce, feed and multiply.
Nanoarchaeon	Very small archaea (measuring between 150 and 500 nm) are characterized by their reduced genome and limited biosynthetic and metabolic capacities, and this organism depends on exo-symbiotic or parasitic interactions with other archaea. Nano-archaea were detected for the first time by metagenomics.
ORFans	These genes have unknown evolutionary identification and origin unique to a given species. The genes may be newly created, generated by the fusion of several sequences, or produced by sequence degradation.
Parasitism	This function is carried out by an organism that is dependent on a host to develop and exploits the host to multiply, feed and reproduce. Parasitism exerts a negative effect on the host.
Rhizome	Rhizomes are a representation of the evolutionary history of a genome based on the totality of its sequence; in contrast, phylogenetic trees are based on single genes. Rhizomes consider sequences resulting from such phenomena as transfers, recombination, fusions, degradation, and de novo creation. Rhizomes consider ORFans, as well.
Symbiont	These organisms need other organisms to live and multiply.
Symbiosis	In this interaction, two organisms live together, and both benefit from the association.
TRUC	An abbreviation of Things Resisting Uncompleted Classification: A new term (which is French for stuff) coined in 2013 to designate major groups of microbes (bacteria, archaea, eukaryota and giant viruses) other than based on ribosomal genes (which define domains). TRUC was first used to designate giant viruses.
Tree of Life	This phylogenetic representation groups together currently living cells and resembles a tree with branches and nodes. This model is currently a matter of debate.
Virus (classical)	Viruses are compulsory parasites that are unable to multiply independently, ultra-filterable using filters with 0.2 µm pore sizes, and not visible by light microscopy. The viral genome is composed of either DNA or RNA, encodes capsid protein(s) but lacks genes encoding translation machinery and an energy production system, as well as ribosomal genes. Viruses reproduce from their nucleic acid inside the host cell only.

## Data Availability

Not applicable.

## Supplementary Materials

The following are available online at https://www.mdpi.com/article/10.3390/ijms22115643/s1, Supplementary Data 1: Hierarchical clustering by phyletic pattern based on the presence/absence of translational COGs, informational COGs and fold proteins superfamily; Supplementary Data 2: Phylogenetic tree of the most conserved genes of giant viruses and the other domains; Table S1: all genomes used in this study.

## References

[1] The Logic of Scientific Discovery—Karl Popper—Google Books. https://books.google.fr/books?hl=en&lr=&id=LWSBAgAAQBAJ&oi=fnd&pg=PP1&dq=popper+1934+the+logic+of+scientific+discovery&ots=pADe_00FdN&sig=rmw7bfCRO3OobPdGEpdjkLaMXBk&redir_esc=y#v=onepage&q=popper1934thelogicofscientificdiscovery&f=false.

[2] Raoult D. (2013). TRUC or the need for a new microbial classification. Intervirology.

[3] Blevins S.M., Bronze M.S. (2010). Robert Koch and the “golden age” of bacteriology. Int. J. Infect. Dis..

[4] Coico R. (2005). Gram staining. Curr. Protoc. Microbiol..

[5] Ivanowski D. (1892). Ueber die Mosaikkrankheit der Tabakspflanze. St. Petersb. Acad Imp. Sci. Bul..

[6] Bos L. (1999). Beijerinck’s work on tobacco mosaic virus: Historical context and legacy. Philos. Trans. R. Soc. Lond. B Biol. Sci..

[7] Wilkinson L. (1974). 1. Beginnings At The Turn Of The Century. Med. Hist..

[8] Lwoff B.A. (1957). The Concept of Virus The Third Marjory Stephenson Memorial Lecture. J. Gen. Microbiol..

[9] Chatton E. (1925). Pansporella Perplexa: Amœbien à Spores Protégées Parasite des Daphnies: Réflexions sur la Biologie et la Phylogénie des Protozoaires.

[10] Smith K.C.A., Wells O.C., Mcmullan D. (2008). The fiftieth anniversary of the first applications of the scanning electron microscope in materials research. Phys. Procedia.

[11] History of Electron Microscopy, 1931–2000. https://authors.library.caltech.edu/5456/1/hrst.mit.edu/hrs/materials/public/ElectronMicroscope/EM_HistOverview.htm.

[12] Woese C.R., Kandler O., Wheelis M.L. (1990). Towards a natural system of organisms: Proposal for the domains Archaea, Bacteria, and Eucarya. Proc. Natl. Acad. Sci. USA.

[13] Lindsay M.R., Webb R.I., Strous M., Jetten M.S.M., Butler M.K., Forde R.J., Fuerst J.A. (2001). Cell compartmentalisation in planctomycetes: Novel types of structural organisation for the bacterial cell. Arch. Microbiol..

[14] Raoult D., Forterre P. (2008). Redefining viruses: Lessons from Mimivirus. Nat. Rev. Microbiol..

[15] Georgiades K., Raoult D. (2011). The rhizome of *Reclinomonas americana*, *Homo sapiens*, *Pediculus humanus* and *Saccharomyces cerevisiae* mitochondria. Biol. Direct.

[16] La Scola B., Audic S., Robert C., Jungang L., De Lamballerie X., Drancourt M., Birtles R., Claverie J.M., Raoult D. (2003). A giant virus in amoebae. Science.

[17] Raoult D., Audic S., Robert C., Abergel C., Renesto P., Ogata H., La Scola B., Suzan M., Claverie J.M. (2004). The 1.2-megabase genome sequence of Mimivirus. Science.

[18] Zaremba-Niedzwiedzka K., Caceres E.F., Saw J.H., Bäckström D., Juzokaite L., Vancaester E., Seitz K.W., Anantharaman K., Starnawski P., Kjeldsen K.U. (2017). Asgard archaea illuminate the origin of eukaryotic cellular complexity. Nature.

[19] Brown C.T., Hug L.A., Thomas B.C., Sharon I., Castelle C.J., Singh A., Wilkins M.J., Wrighton K.C., Williams K.H., Banfield J.F. (2015). Unusual biology across a group comprising more than 15% of domain Bacteria. Nature.

[20] Hug L.A., Baker B.J., Anantharaman K., Brown C.T., Probst A.J., Castelle C.J., Butterfield C.N., Hernsdorf A.W., Amano Y., Ise K. (2016). A new view of the tree of life. Nat. Microbiol..

[21] Soro V., Dutton L.C., Sprague S.V., Nobbs A.H., Ireland A.J., Sandy J.R., Jepson M.A., Micaroni M., Splatt P.R., Dymock D. (2014). Axenic culture of a candidate division TM7 bacterium from the human oral cavity and biofilm interactions with other oral bacteria. Appl. Environ. Microbiol..

[22] Dombrowski N., Lee J.-H., Williams T.A., Offre P., Spang A. (2019). Genomic diversity, lifestyles and evolutionary origins of DPANN archaea. FEMS Microbiol. Lett..

[23] Baker B.J., Comolli L.R., Dick G.J., Hauser L.J., Hyatt D., Dill B.D., Land M.L., VerBerkmoes N.C., Hettich R.L., Banfield J.F. (2010). Enigmatic, ultrasmall, uncultivated Archaea. Proc. Natl. Acad. Sci. USA.

[24] Deschamps P., Zivanovic Y., Moreira D., Rodriguez-Valera F., Lopez-García P. (2014). Pangenome evidence for extensive interdomain horizontal transfer affecting lineage coreandshell genes inuncultured planktonic thaumarchaeota and euryarchaeota. Genome Biol. Evol..

[25] Merhej V., Raoult D. (2012). Rhizome of life, catastrophes, sequence exchanges, gene creations, and giant viruses: How microbial genomics challenges Darwin. Front. Cell. Infect. Microbiol..

[26] Raoult D. (2010). The post-Darwinist rhizome of life. Lancet.

[27] Caetano-Anollés G., Caetano-Anollés D. (2003). An evolutionarily structural universe of protein architecture. Genome Res..

[28] Dagan T., Martin W. (2006). The tree of one percent. Genome Biol..

[29] Deleuze G. (1976). Rhizome: Introduction.

[30] Merhej V., Notredame C., Royer-Carenzi M., Pontarotti P., Raoult D. (2011). The rhizome of life: The sympatric *Rickettsia felis* paradigm demonstrates the random transfer of DNA sequences. Mol. Biol. Evol..

[31] Levasseur A., Merhej V., Baptiste E., Sharma V., Pontarotti P., Raoult D. (2017). The rhizome of *lokiarchaeota* illustrates the mosaicity of archaeal genomes. Genome Biol. Evol..

[32] Darwin C. (2009). CLASSICS On the Origin of Species by Means of Natural Selection, or the Preservation of Favoured Races in the Struggle for Life.

[33] Raoult D., Koonin E.V. (2012). Microbial genomics challenge Darwin. Front. Cell. Infect. Microbiol..

[34] Woese C.R. (1987). Bacterial evolution. Microbiol. Rev..

[35] Woese C.R., Fox G.E. (1977). Phylogenetic structure of the prokaryotic domain: The primary kingdoms. Proc. Natl. Acad. Sci. USA.

[36] Bapteste E., Boucher Y. (2008). Lateral gene transfer challenges principles of microbial systematics. Trends Microbiol..

[37] Baumgartner M., Roffler S., Wicker T., Pernthaler J. (2017). Letting go: Bacterial genome reduction solves the dilemma of adapting to predation mortality in a substrate-restricted environment. ISME J..

[38] Georgiades K., Raoult D. (2012). How microbiology helps define the rhizome of life. Front. Cell. Infect. Microbiol..

[39] Gogarten J.P., Doolittle W.F., Lawrence J.G. (2002). Prokaryotic evolution in light of gene transfer. Mol. Biol. Evol..

[40] Koonin E.V., Puigbò P., Wolf Y.I. (2011). Comparison of phylogenetic trees and search for a central trend in the “forest of life”. J. Comput. Biol..

[41] Penny D. (2011). Darwin’s Theory of Descent with Modification, versus the Biblical Tree of Life. PLoS Biol..

[42] Robbens S., Derelle E., Ferraz C., Wuyts J., Moreau H., Van De Peer Y. (2007). The Complete Chloroplast and Mitochondrial DNA Sequence of Ostreococcus tauri: Organelle Genomes of the Smallest Eukaryote Are Examples of Compaction. Mol. Biol. Evol..

[43] Colson P., Levasseur A., La Scola B., Sharma V., Nasir A., Pontarotti P., Caetano-Anollés G., Raoult D. (2018). Ancestrality and mosaicism of giant viruses supporting the definition of the fourth TRUC of microbes. Front. Microbiol..

[44] Nelson-Sathi S., Sousa F.L., Roettger M., Lozada-Chávez N., Thiergart T., Janssen A., Bryant D., Landan G., Schönheit P., Siebers B. (2015). Origins of major archaeal clades correspond to gene acquisitions from bacteria. Nature.

[45] Da Cunha V., Gaia M., Nasir A., Forterre P. (2018). Asgard archaea do not close the debate about the universal tree of life topology. PLOS Genet..

[46] Liu Y., Makarova K.S., Huang W.C., Wolf Y.I., Nikolskaya A., Zhang X., Cai M., Zhang C.J., Xu W., Luo Z. (2020). Expanding diversity of asgard archaea and the elusive ancestry of eukaryotes. bioRxiv.

[47] Macleod F., Kindler G.S., Wong H.L., Chen R., Burns B.P. (2019). Asgard archaea: Diversity, function, and evolutionary implications in a range of microbiomes. AIMS Microbiol..

[48] Koonin E.V., Yutin N. (2014). The dispersed archaeal eukaryome and the complex archaeal ancestor of eukaryotes. Cold Spring Harb. Perspect. Biol..

[49] López-García P., Moreira D. (2020). Cultured Asgard Archaea Shed Light on Eukaryogenesis. Cell.

[50] Karnkowska A., Vacek V., Zubáčová Z., Treitli S.C., Petrželková R., Eme L., Novák L., Žárský V., Barlow L.D., Herman E.K. (2016). A eukaryote without a mitochondrial organelle. Curr. Biol..

[51] López-García P., Moreira D. (2020). The Syntrophy hypothesis for the origin of eukaryotes revisited. Nat. Microbiol..

[52] Falcón L.I., Magallón S., Castillo A. (2010). Dating the cyanobacterial ancestor of the chloroplast. ISME J..

[53] Doolittle W.F., Bapteste E. (2007). Pattern pluralism and the Tree of Life hypothesis. Proc. Natl. Acad. Sci. USA.

[54] Eme L., Spang A., Lombard J., Stairs C.W., Ettema T.J.G. (2018). Erratum: Archaea and the origin of eukaryotes (Nature reviews. Microbiology (2017) 15 12 (711–723)). Nat. Rev. Microbiol..

[55] Eme L., Spang A., Lombard J., Stairs C.W., Ettema T.J.G. (2017). Archaea and the origin of eukaryotes. Nat. Rev. Microbiol..

[56] Forterre P., Gaïa M. (2016). Giant viruses and the origin of modern eukaryotes. Curr. Opin. Microbiol..

[57] Feschotte C., Gilbert C. (2012). Endogenous viruses: Insights into viral evolution and impact on host biology. Nat. Rev. Genet..

[58] Nikoh N., Tanaka K., Shibata F., Kondo N., Hizume M., Shimada M., Fukatsu T. (2008). *Wolbachia* genome integrated in an insect chromosome: Evolution and fate of laterally transferred endosymbiont genes. Genome Res..

[59] Callaway E. (2007). Genomes within genomes. Nature.

[60] Arbuckle J.H., Medveczky M.M., Luka J., Hadley S.H., Luegmayr A., Ablashi D., Lund T.C., Tolar J., De Meirleir K., Montoya J.G. (2010). The latent human herpesvirus-6A genome specifically integrates in telomeres of human chromosomes in vivo and in vitro. Proc. Natl. Acad. Sci. USA.

[61] Abi-Rached L., Jobin M.J., Kulkarni S., McWhinnie A., Dalva K., Gragert L., Babrzadeh F., Gharizadeh B., Luo M., Plummer F.A. (2011). The shaping of modern human immune systems by multiregional admixture with archaic humans. Science.

[62] Lacroix B., Citovsky V. (2016). Transfer of DNA from bacteria to eukaryotes. mBio.

[63] Brueckner J., Martin W.F. (2020). Bacterial Genes Outnumber Archaeal Genes in Eukaryotic Genomes. Genome Biol. Evol..

[64] Stoye J.P. (2006). Koala retrovirus: A genome invasion in real time. Genome Biol..

[65] Moniruzzaman M., Weinheimer A.R., Martinez-Gutierrez C.A., Aylward F.O. (2020). Widespread endogenization of giant viruses shapes genomes of green algae. Nature.

[66] Schvarcz C.R., Steward G.F. (2018). A giant virus infecting green algae encodes key fermentation genes. Virology.

[67] Moniruzzaman M., Martinez-Gutierrez C.A., Weinheimer A.R., Aylward F.O. (2020). Dynamic genome evolution and complex virocell metabolism of globally-distributed giant viruses. Nat. Commun..

[68] Filée J. (2014). Multiple occurrences of giant virus core genes acquired by eukaryotic genomes: The visible part of the iceberg?. Virology.

[69] Chelkha N., Hasni I., Louazani A.C., Levasseur A., Scola B. (2020). La *Vermamoeba vermiformis* CDC-19 draft genome sequence reveals considerable gene trafficking including with candidate phyla radiation and giant viruses. Sci. Rep..

[70] Balczun C., Scheid P. (2017). Free-Living Amoebae as Hosts for and Vectors of Intracellular Microorganisms with Public Health Significance. Viruses.

[71] Maumus F., Quesneville H. (2014). Ancestral repeats have shaped epigenome and genome composition for millions of years in Arabidopsis thaliana. Nat. Commun..

[72] Chelkha N., Levasseur A., Pontarotti P., Raoult D., La Scola B., Colson P. (2018). A Phylogenomic Study of Acanthamoeba polyphaga Draft Genome Sequences Suggests Genetic Exchanges With Giant Viruses. Front. Microbiol..

[73] Blanc G., Gallot-Lavallée L., Maumus F. (2015). Provirophages in the *Bigelowiella* genome bear testimony to past encounters with giant viruses. Proc. Natl. Acad. Sci. USA.

[74] Fischer M.G., Hackl T. (2016). Host genome integration and giant virus-induced reactivation of the virophage mavirus. Nature.

[75] Aherfi S., Belhaouari D.B., Pinault L., Baudoin J.P., Decloquement P., Abrahao J., Colson P., Levasseur A., Lamb D.C., Chabriere E. (2020). Tricarboxylic acid cycle and proton gradient in Pandoravirus massiliensis: Is it still a virus?. bioRxiv.

[76] Fischer M.G., Allen M.J., Wilson W.H., Suttle C.A. (2010). Giant virus with a remarkable complement of genes infects marine zooplankton. Proc. Natl. Acad. Sci. USA.

[77] Deeg C.M., Chow C.E.T., Suttle C.A. (2018). The kinetoplastid-infecting bodo saltans virus (Bsv), a window into the most abundant giant viruses in the sea. eLife.

[78] Boyer M., Madoui M.-A., Gimenez G., La Scola B., Raoult D. (2010). Phylogenetic and Phyletic Studies of Informational Genes in Genomes Highlight Existence of a 4th Domain of Life Including Giant Viruses. PLoS ONE.

[79] Fraser C.M., Eisen J.A., Salzberg S.L. (2000). Microbial genome sequencing. Nature.

[80] Bordenstein S.R., Reznikoff W.S. (2005). Mobile DNA in obligate intracellular bacteria. Nat. Rev. Microbiol..

[81] Ogata H., La Scola B., Audic S., Renesto P., Blanc G., Robert C., Fournier P.-E., Claverie J.-M., Raoult D. (2006). Genome Sequence of *Rickettsia bellii* Illuminates the Role of Amoebae in Gene Exchanges between Intracellular Pathogens. PLoS Genet..

[82] Shintani M. (2017). The behavior of mobile genetic elements (MGEs) in different environments. Biosci. Biotechnol. Biochem..

[83] Ochman H., Lerat E., Daubin V. (2005). Examining bacterial species under the specter of gene transfer and exchange. Proc. Natl. Acad. Sci. USA.

[84] Dokland T. (2019). Molecular Piracy: Redirection of Bacteriophage Capsid Assembly by Mobile Genetic Elements. Viruses.

[85] Bitto N.J., Chapman R., Pidot S., Costin A., Lo C., Choi J., D’Cruze T., Reynolds E.C., Dashper S.G., Turnbull L. (2017). Bacterial membrane vesicles transport their DNA cargo into host cells. Sci. Rep..

[86] Anderson M.T., Seifert H.S. (2011). Opportunity and means: Horizontal gene transfer from the human host to a bacterial pathogen. mBio.

[87] Castelle C.J., Brown C.T., Anantharaman K., Probst A.J., Huang R.H., Banfield J.F. (2018). Biosynthetic capacity, metabolic variety and unusual biology in the CPR and DPANN radiations. Nat. Rev. Microbiol..

[88] Shao R., Kirkness E.F., Barker S.C. (2009). The single mitochondrial chromosome typical of animals has evolved into 18 minichromosomes in the human body louse, *Pediculus humanus*. Genome Res..

[89] Esser C., Martin W., Dagan T. (2007). The origin of mitochondria in light of a fluid prokaryotic chromosome model. Biol. Lett..

[90] Deusch O., Landan G., Roettger M., Gruenheit N., Kowallik K.V., Allen J.F., Martin W., Dagan T. (2008). Genes of cyanobacterial origin in plant nuclear genomes point to a heterocyst-forming plastid ancestor. Mol. Biol. Evol..

[91] Timmis J.N., Ayliff M.A., Huang C.Y., Martin W. (2004). Endosymbiotic gene transfer: Organelle genomes forge eukaryotic chromosomes. Nat. Rev. Genet..

[92] Zhaxybayeva O., Gogarten J.P. (2004). Cladogenesis, coalescence and the evolution of the three domains of life. Trends Genet..

[93] Peretó J., López-García P., Moreira D. (2004). Ancestral lipid biosynthesis and early membrane evolution. Trends Biochem. Sci..

[94] Viezens J., Arvard M. (2008). Simultaneous presence of two different copies of the 16S rRNA gene in *Bartonella henselae*. Microbiology.

[95] Seemann T. (2014). Prokka: Rapid prokaryotic genome annotation. Bioinformatics.

[96] Buchfink B., Xie C., Huson D.H. (2014). Fast and sensitive protein alignment using DIAMOND. Nat. Methods.

[97] Lechner M., Findeiß S., Steiner L., Marz M., Stadler P.F., Prohaska S.J. (2011). Proteinortho: Detection of (Co-)orthologs in large-scale analysis. BMC Bioinform..

[98] Nasir A., Sun F.-J., Kim K.M., Caetano-Anollés G. (2015). Untangling the origin of viruses and their impact on cellular evolution. Ann. N. Y. Acad. Sci..

[99] Méheust R., Burstein D., Castelle C.J., Banfield J.F. (2019). The distinction of CPR bacteria from other bacteria based on protein family content. Nat. Commun..

[100] Bokhari R.H., Amirjan N., Jeong H., Kim K.M., Caetano-Anollés G., Nasir A., Bapteste E. (2020). Bacterial Origin and Reductive Evolution of the CPR Group. Genome Biol. Evol..

[101] Tian R., Ning D., He Z., Zhang P., Spencer S.J., Gao S., Shi W., Wu L., Zhang Y., Yang Y. (2020). Small and mighty: Adaptation of superphylum *Patescibacteria* to groundwater environment drives their genome simplicity. Microbiome.

[102] Edgar R.C. (2004). MUSCLE: Multiple sequence alignment with high accuracy and high throughput. Nucleic Acids Res..

[103] Sharma V., Colson P., Pontarotti P., Raoult D. (2016). Mimivirus inaugurated in the 21st century the beginning of a reclassification of viruses. Curr. Opin. Microbiol..

[104] Fournier G.P., Poole A.M. (2018). A briefly argued case that Asgard Archaea are part of the eukaryote tree. Front. Microbiol..

[105] Sharma V., Colson P., Chabrol O., Scheid P., Pontarotti P., Raoult D. (2015). Welcome to pandoraviruses at the “Fourth TRUC” club. Front. Microbiol..

[106] Sharma V., Colson P., Chabrol O., Pontarotti P., Raoult D. (2015). Pithovirus sibericum, a new bona fide member of the “Fourth TRUC” club. Front. Microbiol..

[107] Moliner C., Fournier P.-E., Raoult D. (2010). Genome analysis of microorganisms living in amoebae reveals a melting pot of evolution. FEMS Microbiol. Rev..

