# Supplementary material for: Rhizomal Reclassification of Living Organisms
Source: Int J Mol Sci. 2021 May 26;22(11):5643. doi: 10.3390/ijms22115643 (PMC8199106; doi:10.3390/ijms22115643)

Hierarchical clustering by phyletic pattern based on the presence/absence of 180 translational Clusters of Orthologous Groups (COGs) of proteins.

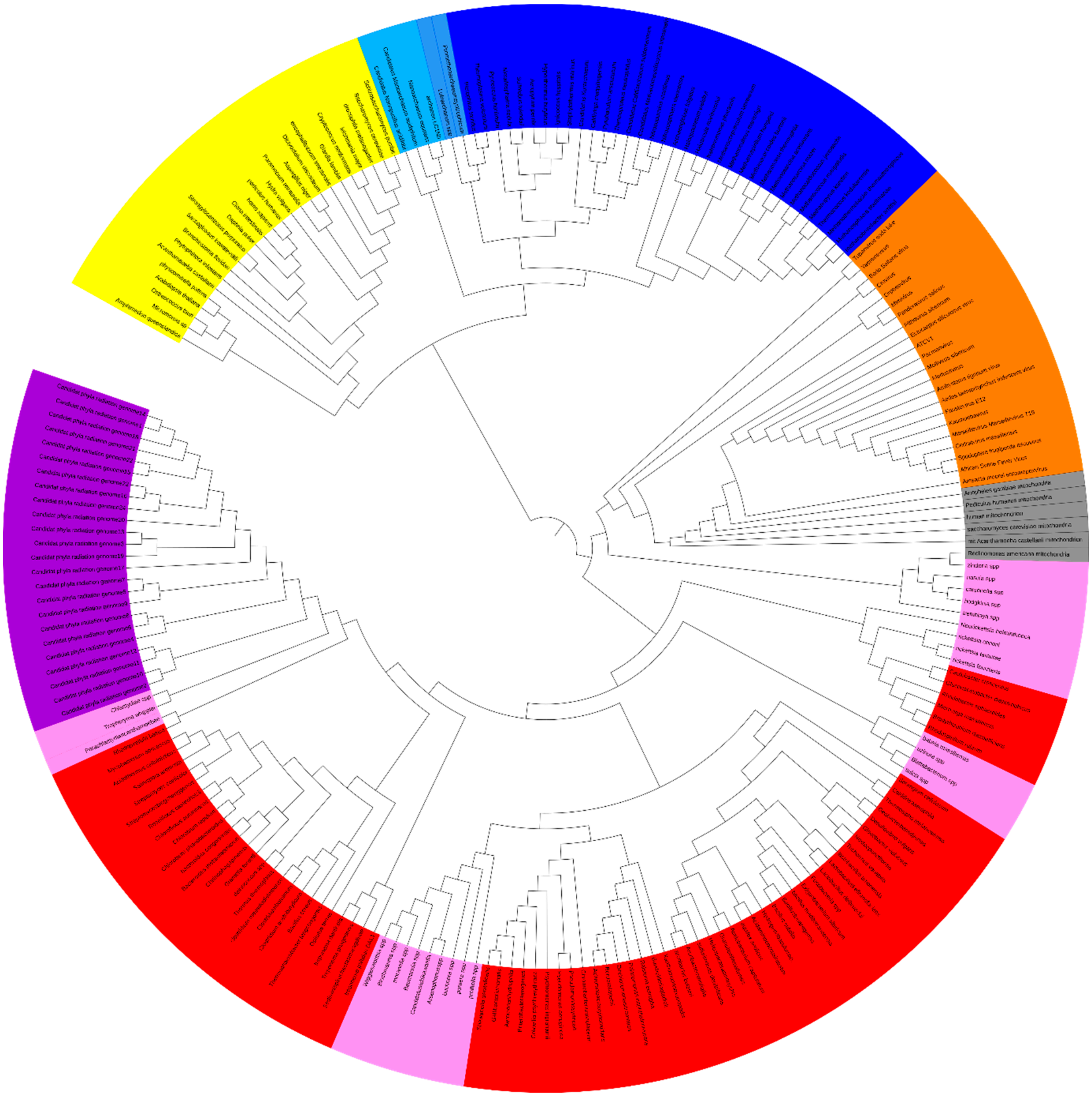

Hierarchical clustering by phyletic pattern based on the presence/absence of 737 informational Clusters of Orthologous Groups (COGs) of proteins

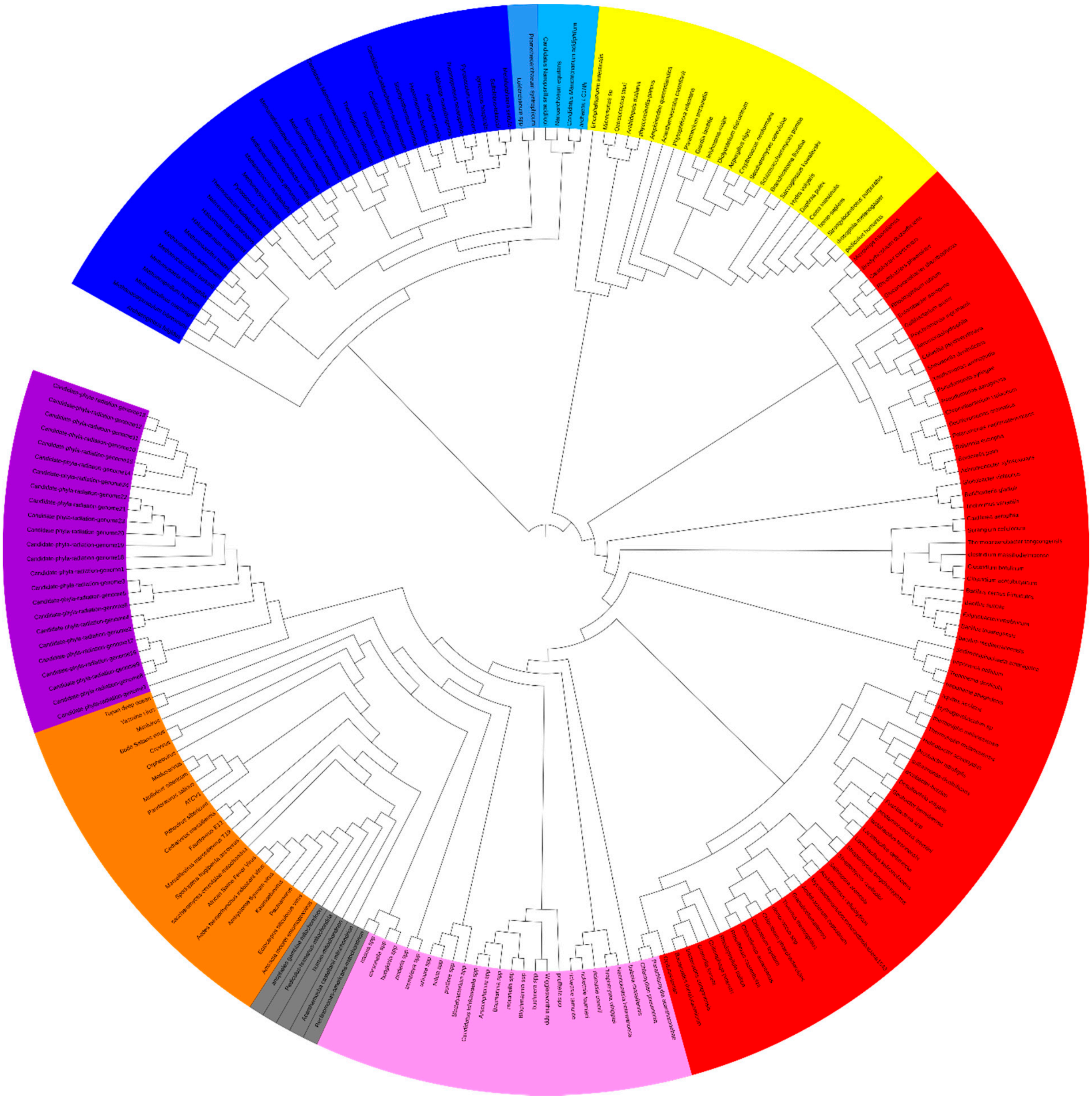

Hierarchical clustering by phyletic pattern based on the presence/absence of 606 fold proteins superfamily.

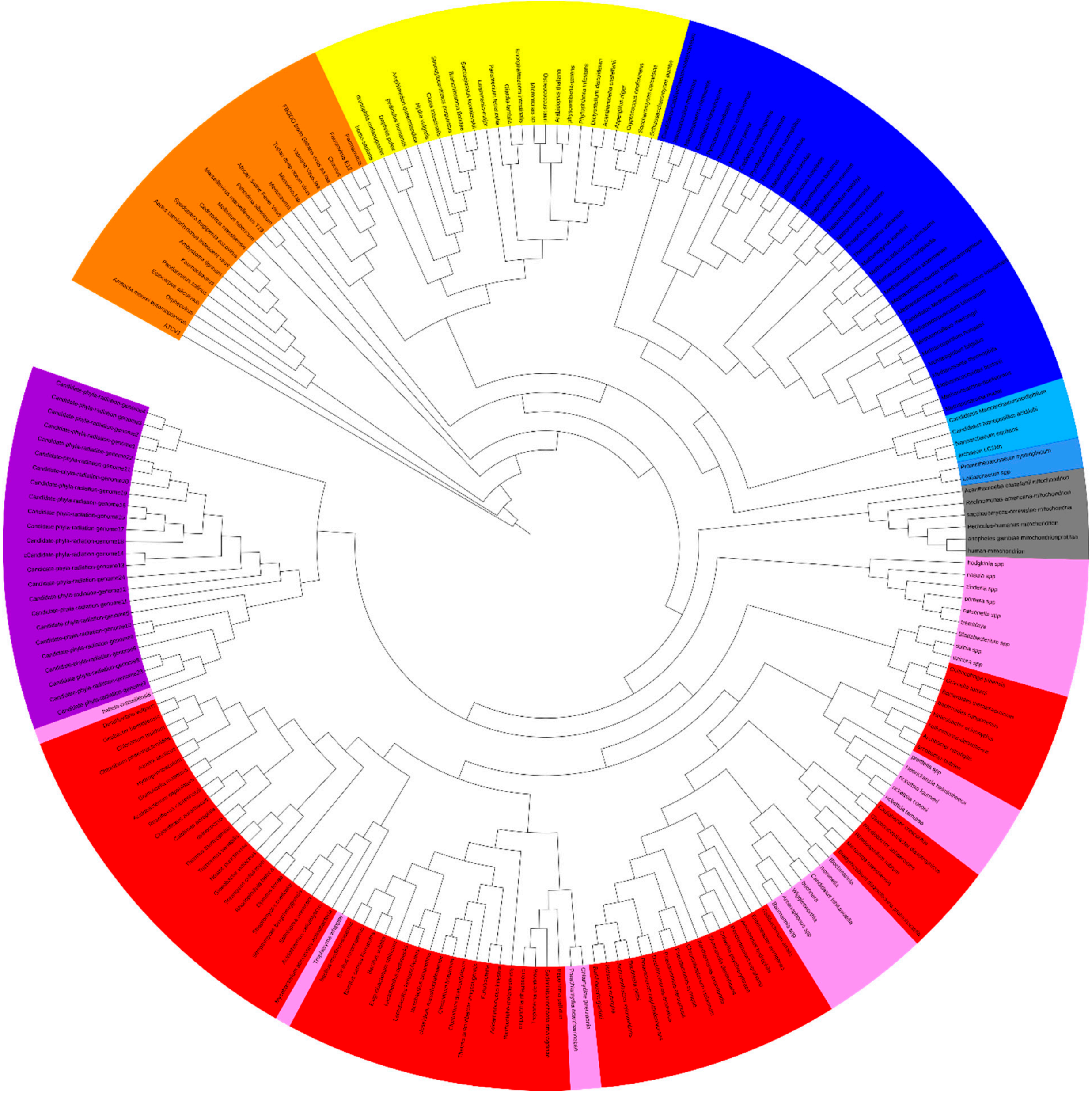

Supplement: Supplementary file 1 [file ijms-22-05643-s001.zip › Supplementary_data_1_figure_5.pdf]
