# Supplementary material for: Rhizomal Reclassification of Living Organisms
Source: Int J Mol Sci. 2021 May 26;22(11):5643. doi: 10.3390/ijms22115643 (PMC8199106; doi:10.3390/ijms22115643)

Figure 6 A: 16S – 18S rRNA gene

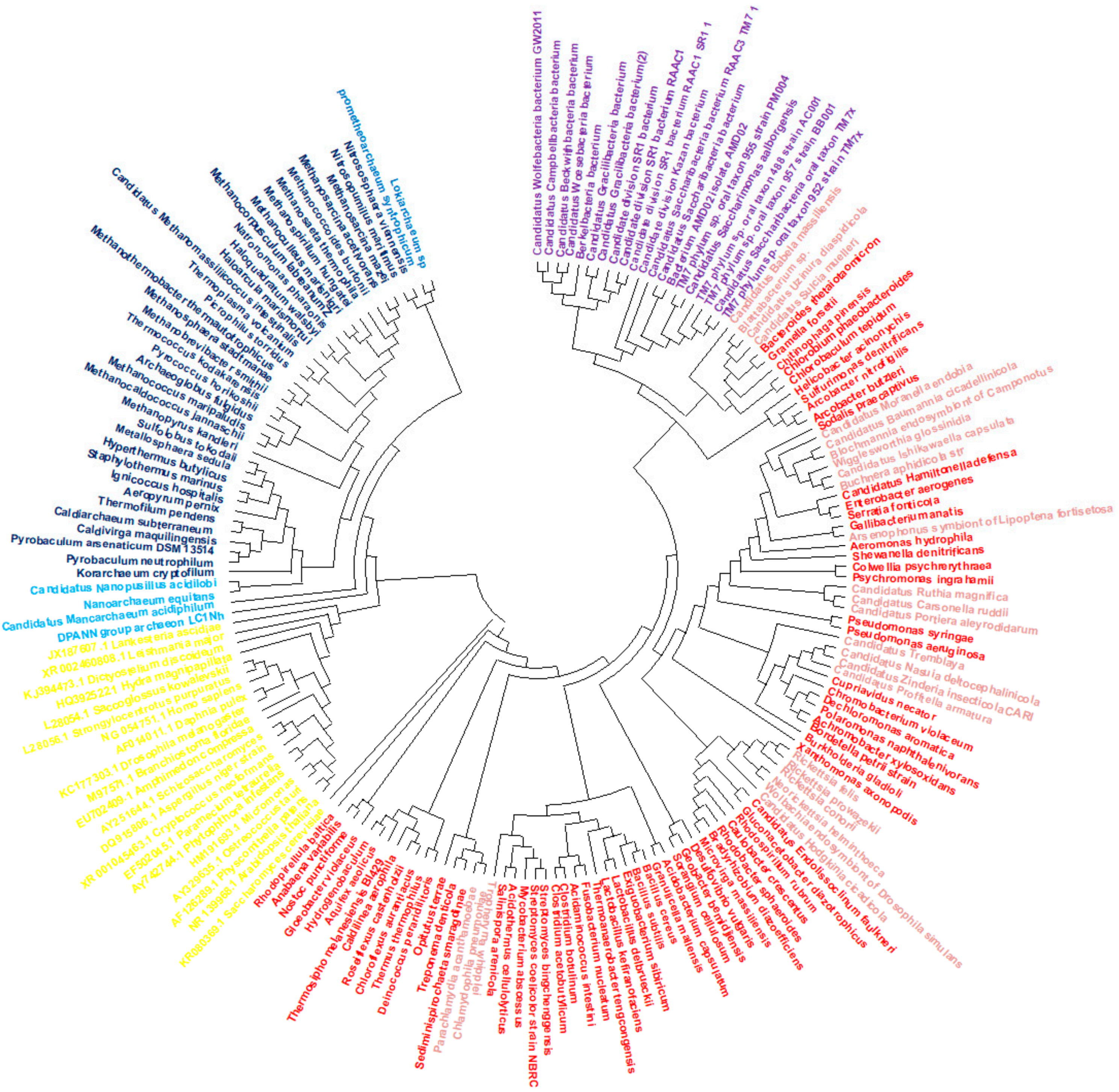

Figure 6 B: 23S – 28S rRNA gene

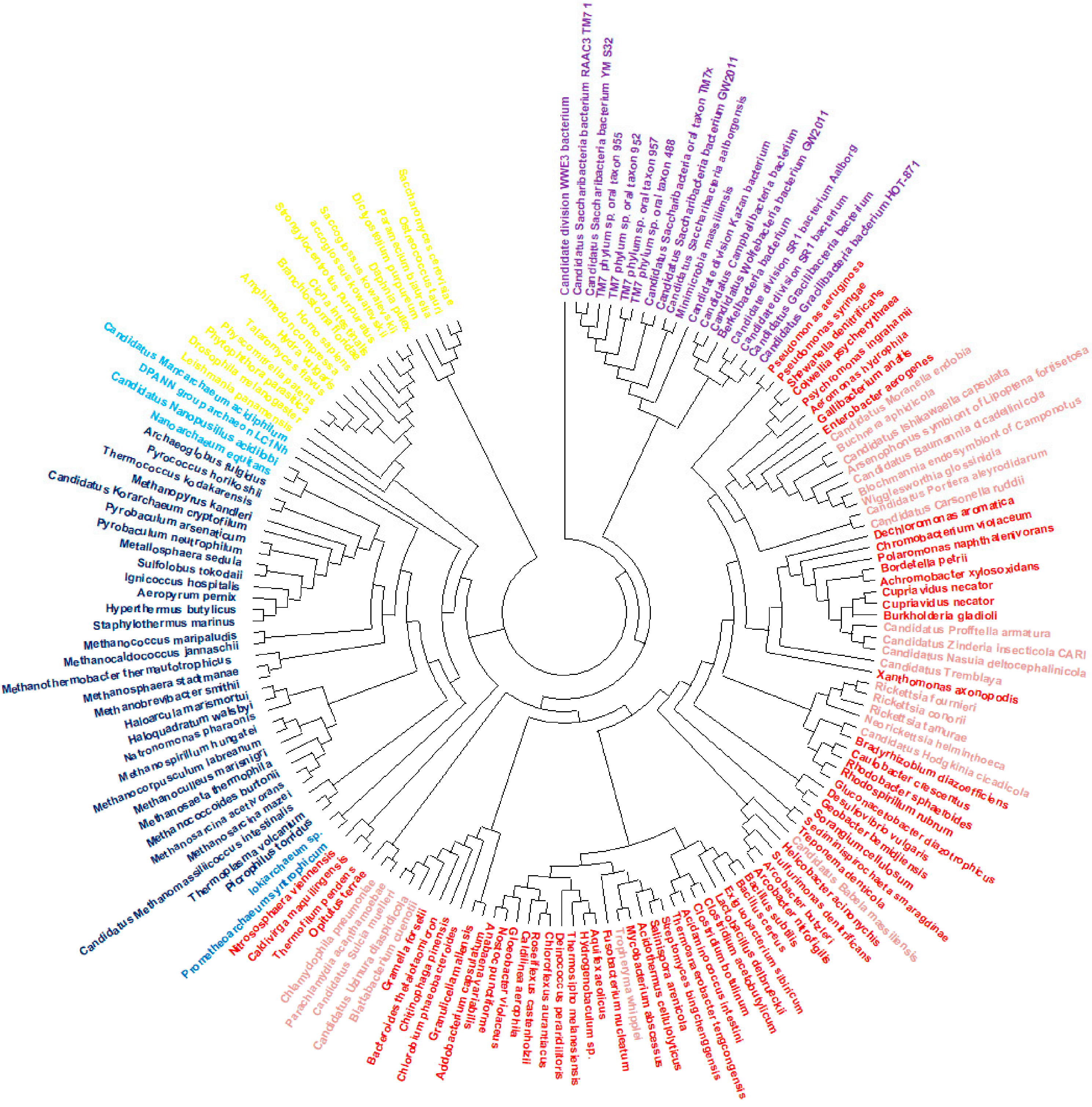

Figure 6 C: DNA Polymerase gene

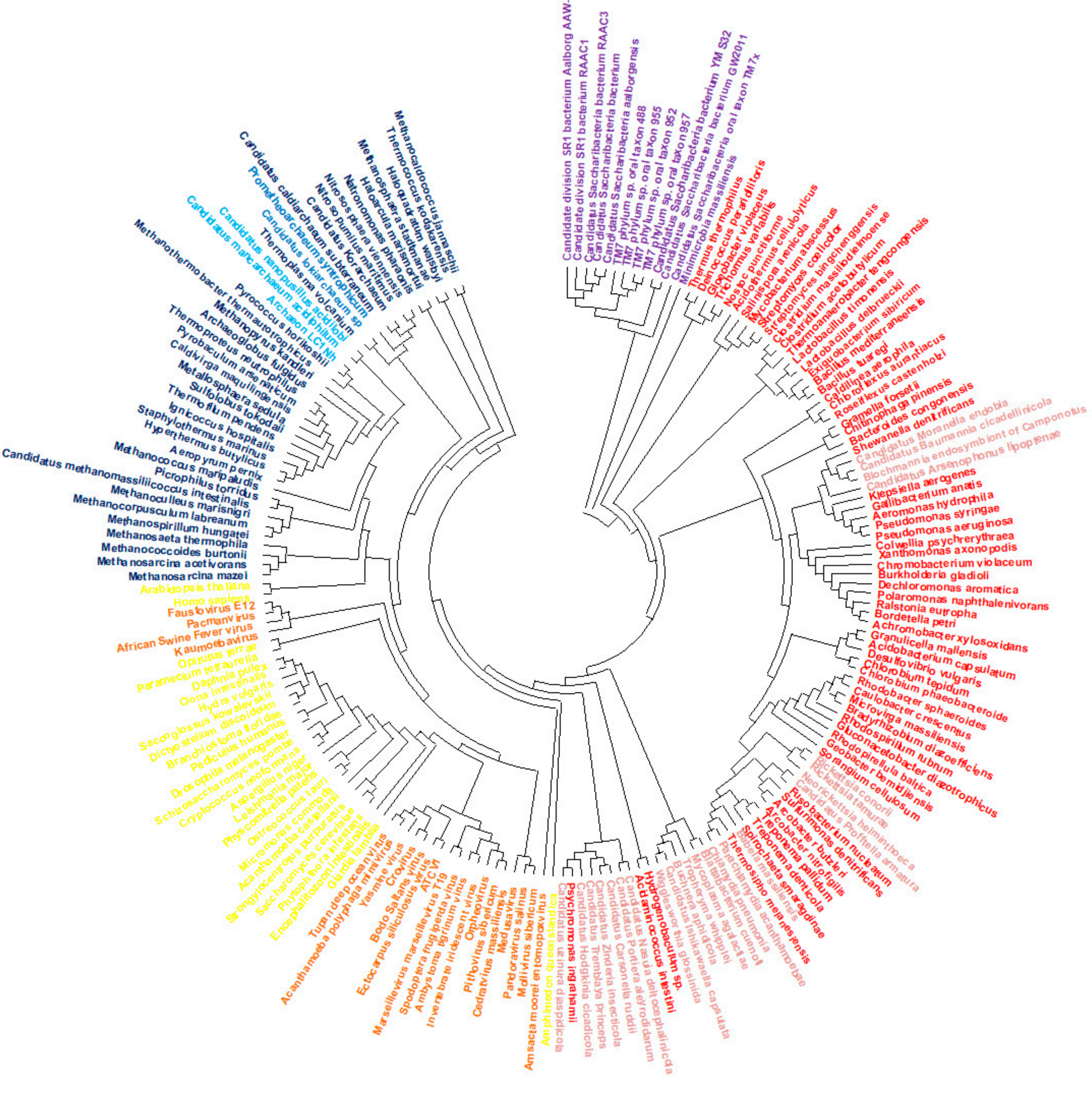

Figure 6 D: DNA-dependent RNA polymerase subunit II

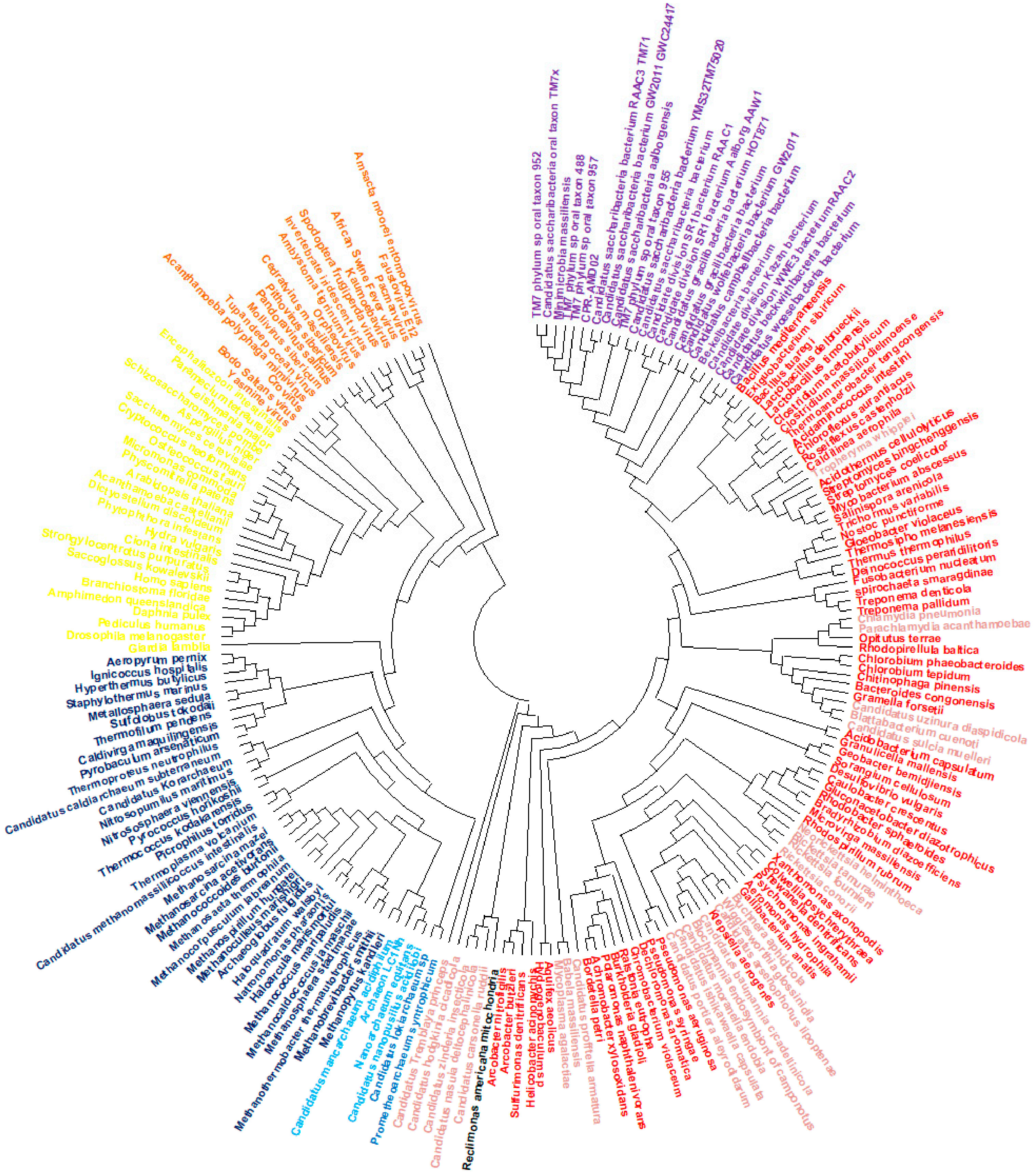

Figure 6 E: Elongation factor I

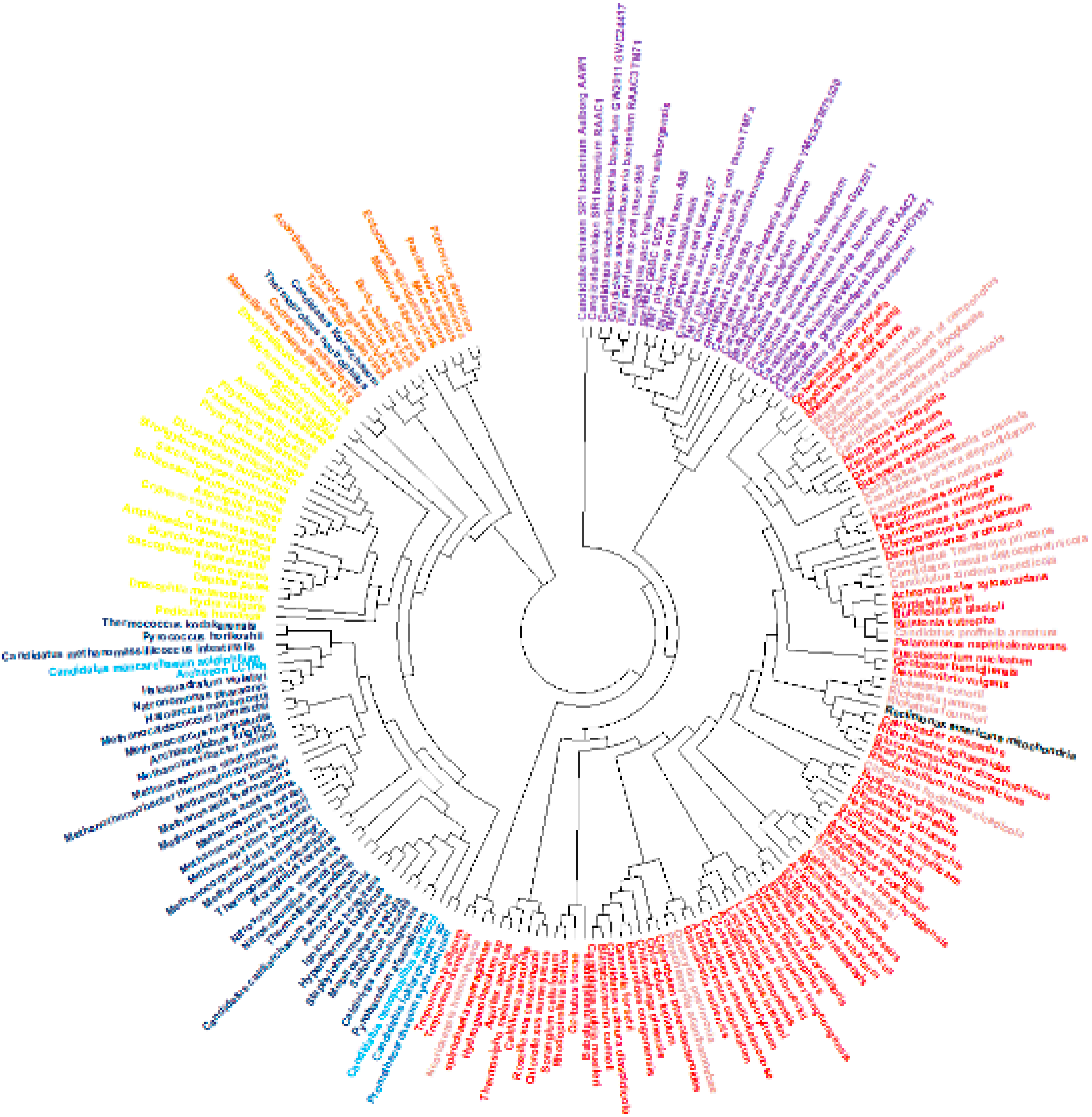

Figure 6 F: Ribonucleotide reductase

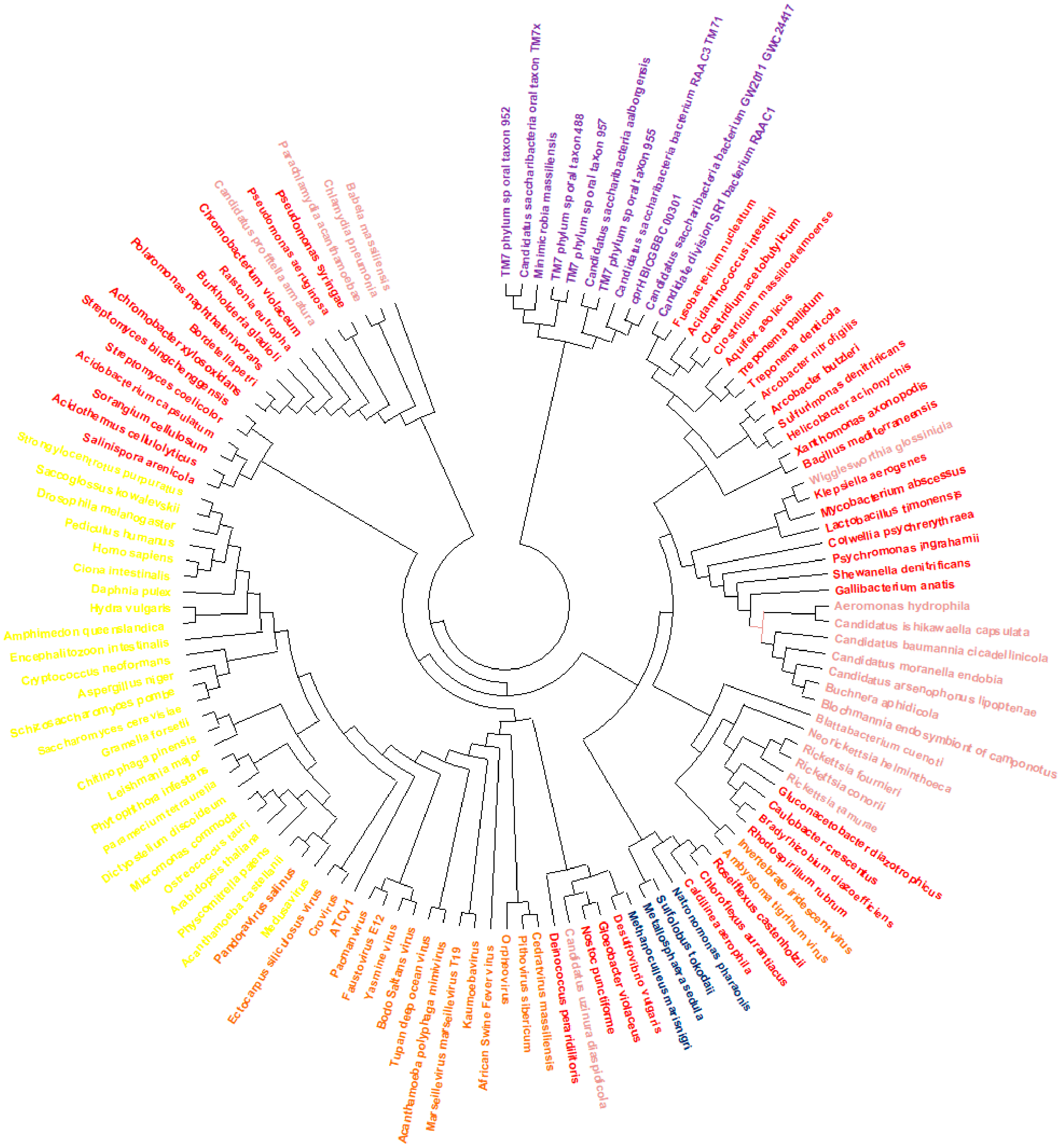

Figure 6 G: Thymidine synthase

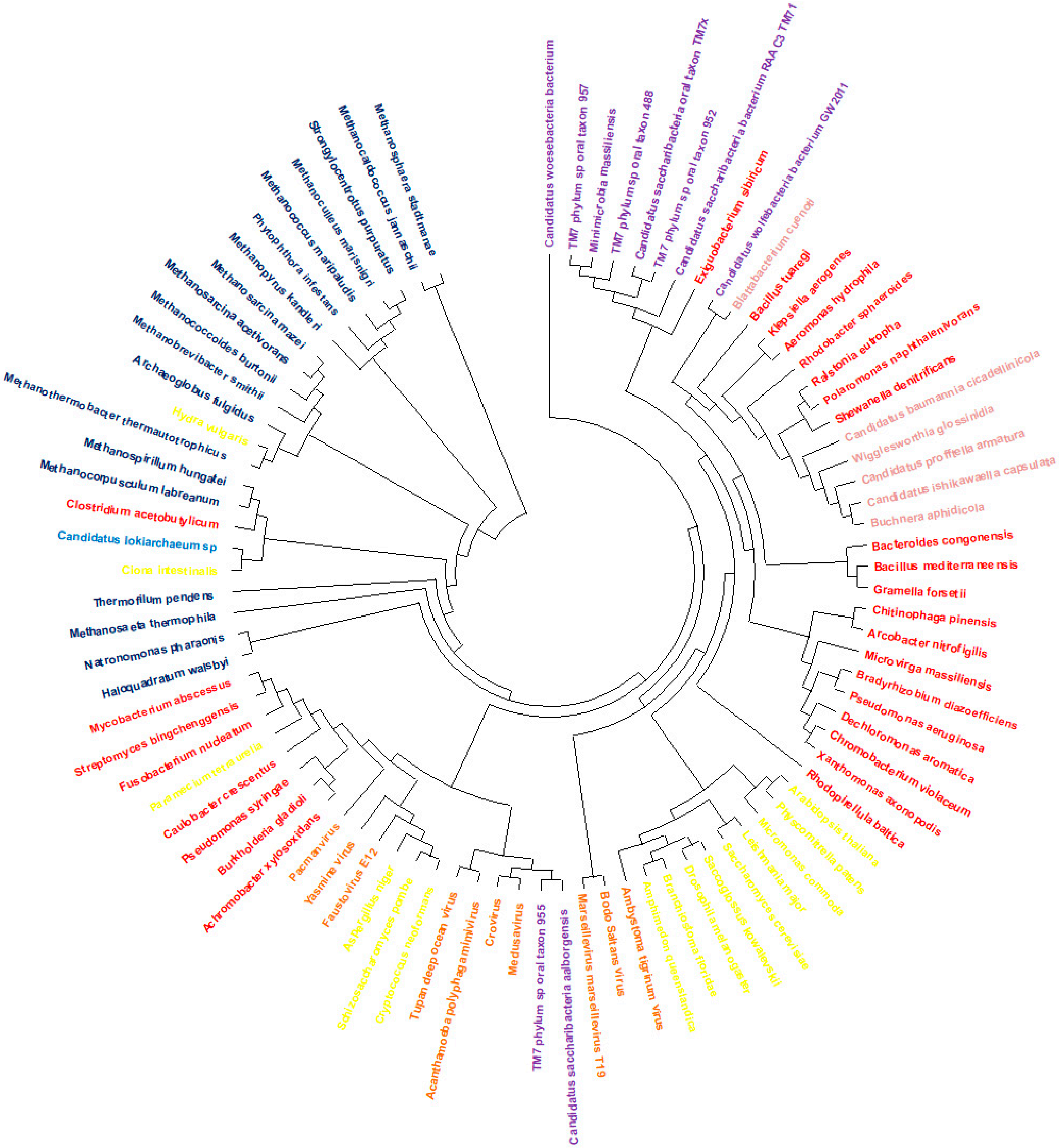

Figure 6 H: Topoisomerase IIA

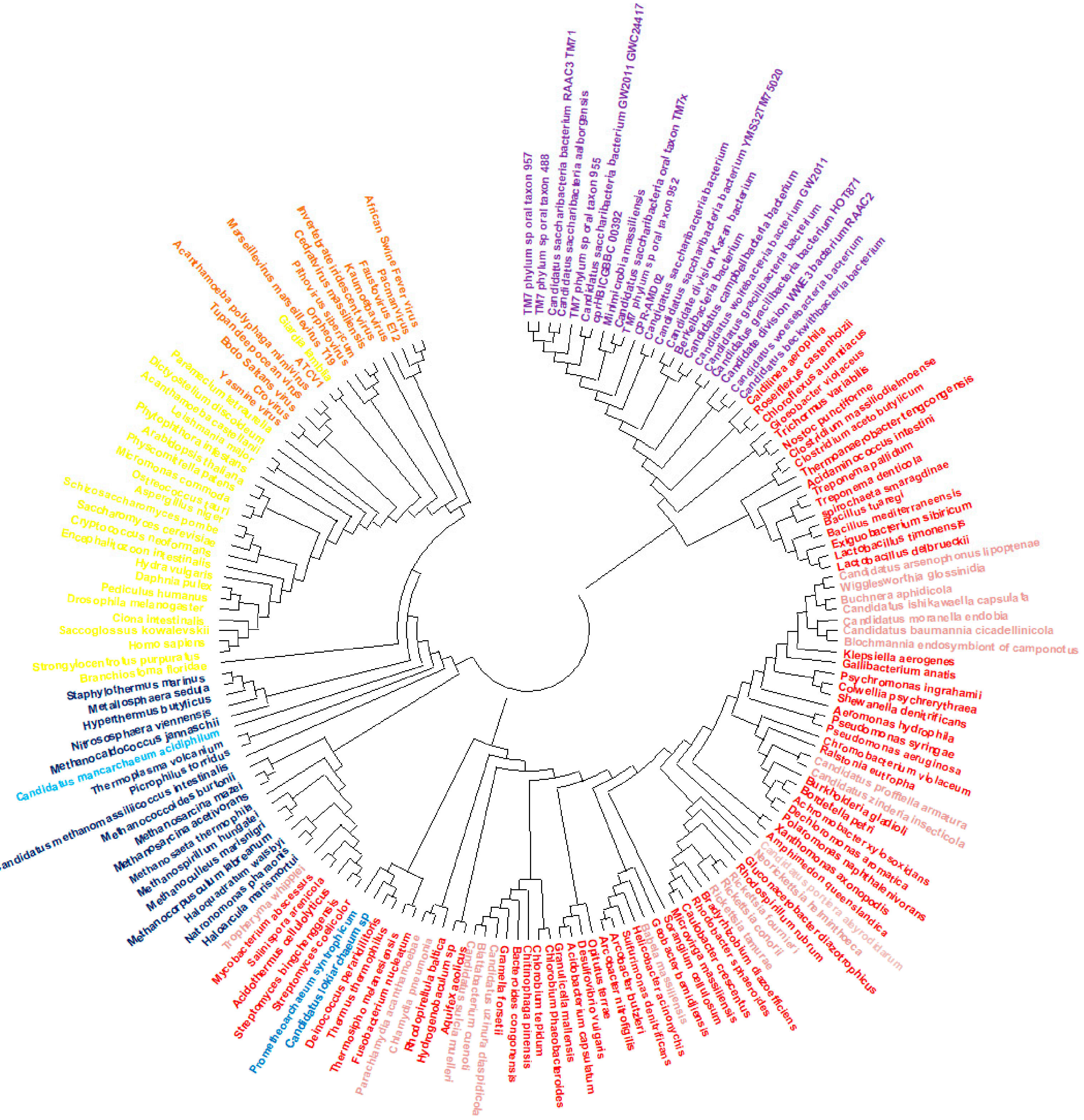

Figure 6 I: Flap endonuclease I

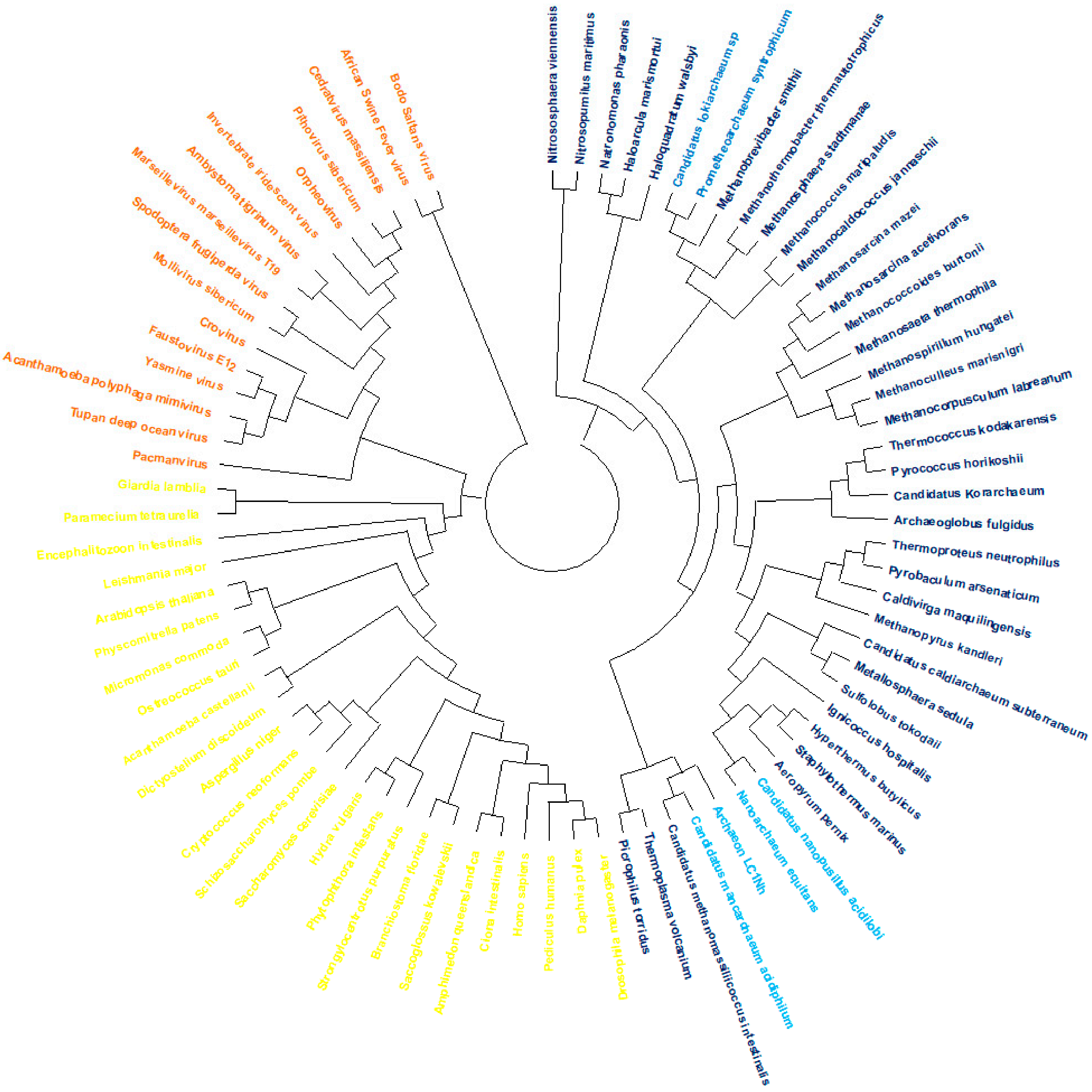

Figure 6 J: Proliferating Cell Nuclear Antigen

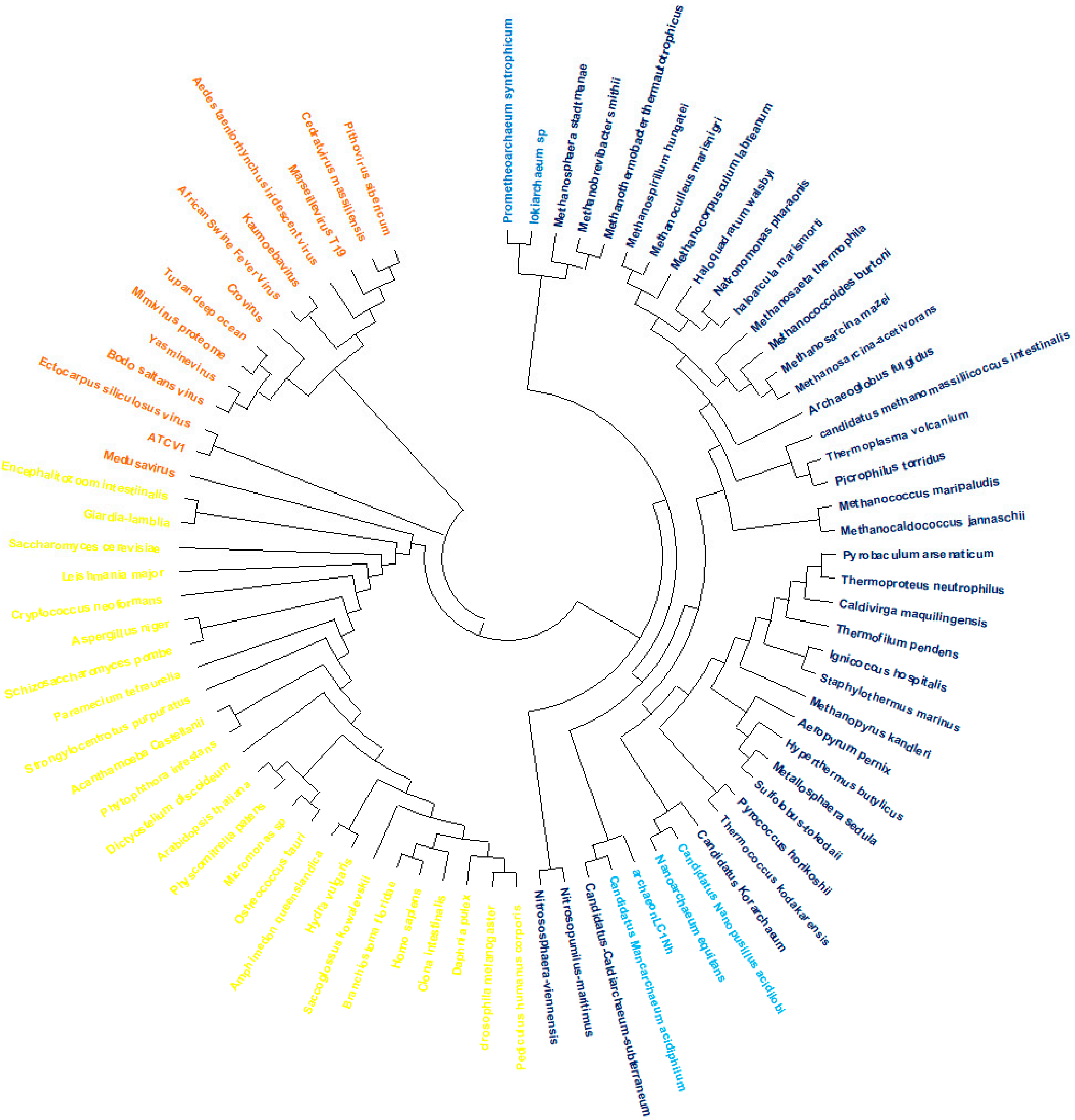

Supplement: Supplementary file 1 [file ijms-22-05643-s001.zip › Supplementary_data_2_Figure_6.pdf]
