# Supplementary material for: Rhizomal Reclassification of Living Organisms
Source: Int J Mol Sci. 2021 May 26;22(11):5643. doi: 10.3390/ijms22115643 (PMC8199106; doi:10.3390/ijms22115643)
Supplement: Supplementary file 1 [file ijms-22-05643-s001.zip › Supplementary_table_1.pdf]

TRUC	Group	Phylum	Accession number
Archae		Euryarchaeotes	AE000782.1
			CP005934.1
			CP039138.1
			FR746099.1
			CP000816.1
			PRJEB30627
			L77117.1
			CP000300.1
			CP000745.1
			CP000559.1
			CP000562.1
			AE009439.1
			CP000477.1
			CP009512.1
			CP000102.1
			CP000254.1
			AE000666.1
			CR936257.1
			AE017261.1
			BA000001.2
			AP006878.1
			BA000011.4
		Crenarchaeotes	BA000002.3
			CP000852.1
			CP000493.1
			CP000682.1
			AE010299.1
			CP000660.1
			CP000575.1
			BA000023.2
			CP001014.1
		Thaumarchaeotes	CP000866.1
			CP007536.1
		Candidatus Korarchaeota	CP000968.1
		Aigarchaeota	BA000048.1
		Asgard Group	ASM98684v1
			CP042905.1
Archae	DPANN group	unclassified DPANN group	CP040089.1
		Micrarchaeota	CP019964.1
		Nanoarchaeota	CP010514.1
			AE017199.1
			v1.0
			ASM285v2

Eukaryote	Opisthokonta	gb|ABEP00000000.2
		KH
		ASM9104v1
		PA42 4.1
		Release 6 plus ISO1 MT
		CP001943.1
		GRCh38.p13
		Hydra_RP_1.0
		JCVI_LOUSE_1.0
		sacCer3
		Skow_1.1
		ASM294v2
		Spur_5.0
	Viridiplantae	TAIR10.1
		ASM9098v2
		version 140606
		Phypa V3
	Stramenopiles	ASM14294v1
	Alveolata	ASM16542v1
	Amoebozoa	Acastellanii.strNEFF v1
		dicty_2.7
	Euglenozoa	ASM272v1
	Metamonada	UU_WB_2.1
Candidate phyla radiation	Kazan	CP011216.1
	Parcubacteria	CP011209.1
		CP011215.1
	Microgenomates	CP011214.1
		CP011210.1
	Katanobacteria	CP006914.1
	Berkelbacteria	CP011213.1
	Saccharibacteria	ASM39243v1
		ASM351602v1
		ASM102969v1
		ASM50391v1
		ASM295275v1
		ASM80362v1
		CADDWL00000000
		ASM569721v1
		ASM1310084v1
		ASM1310082v1
		ASM569739v1
		ASM569705v1
		ASM569756v1
	Absconditabacteria	CP011268.1
		CP006913.1
	Gracilibacteria	CP017714.1

		Gracilibacteria	CP042461.1
	Intracellular bacteria	Bacteriodetes/Chlorobi group	CP003263.1
			AP013293.1
			AP012548.1
		Enterobacteria	AP010872.1
			CP010049.1
			CP003315.1
			CP013920.1
			CP002243.1
			BA000003.2
		Proteobacteria	CP008985.1
			CP003468.1
			CP016343.1
			CP003543.1
			CP008699.1
			CP002918.1
			CP002161.1
			RcoCa1.0
			PRJEB23962
			AT-1
			CP007481.1
			CP021173.1
		Terrabacteria	AE014184.1
		Chlamydia	FR872580.1
			AE001363.1
		Unclassified bacteria	HG793133.1
		Acidobacteria	CP001472.1
			CP003130.1
		Aquificiae	CP001130.1
			AE000657.1
			CP061341.1
		Firmicutes	CR954253.1
			PRJEB20106
			CP003058.1
			AE008691.1
			CP001022.1
			PRJEB20368
			CP028842.1
			AM412317.1
			CP001176.1
			AL009126.3
			PRJEB16343
			PRJEB17078
		Planctomycetes	BX294133.1
		Deinococcus-thermus	AP008226.1
			CP003382.1

Bacteria	Other bacteria phyla	Actinobacteria	CP000481.1
			CU458896.1
			CP000850.1
			AL939104.1
			CP002047
		Bacteriodetes/Chlorobi group	AE006470.1
			CP000492.1
			CU207366.1
			LT598566
			PRJEB18268
			CP001699.1
		Verrucomicrobia	CP001032.1
		Thermotogae	CP000716.1
		Chloroflexi	CP000909.1
			AP012337.1
			CP000804.1
		Cyanobacteria	BA000045.2
			CP000117.1
			CP001037.1
		Proteobacteria	CP000153.1
			CP000361.1
			CP002667.1
			AM260522.1
			CP001999.1
			AE017285.1
			CP001189.1
			CP001340.1
			CP000230.1
			CP000089.1
			CP000302.1
			CP000510.1
			CP000143.2
			CP001124.1
			CP000462.1
			AE016825.1
			AM902716.1
			CP002824.1
			CP004399.1
			CP000529.1
			CP000083.1
			AE016853.1
			AE004091.2
			CP002287.1
			CP002877.1
			CP002599.1
			JC 119
			BA000040.2

			AM746676.1
		Spirochaetes	CP002116.1
			CP003115.1
			CP042818.1
			PRJEB27762
		Fusobacteria	AE009951.2
Giant viruses		Ascoviridae	NC_008361.1
		Asfarvirus	AM712239
		Iridoviridae	NC_005832.1
			NC_008187.1
		Marseilleviridae	ViralProj43573
		Mimiviridae	MF782455.1
			ViralProj59783
			ViralProj60053
			ASM296648v1
			[illegible]SH01000001.1, UPSH0100000[illegible]
		Phycodnaviridae	NC_008724.1
			NC_002687.1
			LT906555.1
		Pithoviridae	NC_032108.1
			NC_023423.1
		Poxviridae	NC_002520.1
		Unclassified viruses	KJ614390.1
			MW018138.1
			KC977571
			NC_027867.1
			NC_034249.1
			LT706986.1
Mitochondria			NC_012920.1
			NC_001823.1
			NC_027264.1
			NC_002084.1
			NC_001637.1
			FJ499473–FJ499490 , FJ514591–FJ514599
			NC_008290.1
Chloroplast			NC_008289.1
			NC_012575.1

Species	Genome size (Kb)	Number of coding genes	Number of COGs	Number of COG - J
Archaeoglobus fulgidus	2,178,400	2407	286	123
Candidatus Methanomassiliicoccus intestinalis	1,931,651	1826	177	92
Haloarcula marismouti	4,184,924	3142	187	89
Haloquadratum walsbyi	3,260,476	2907	184	86
Ignicoccus hospitalis	1,297,538	1434	169	86
Methanobrevibacter smithii	1,853,160	1605	191	91
Methanocaldococcus jannaschii	1,739,927	1715	190	91
Methanococcoides burtonii	2,575,032	2272	196	88
Methanococcus maripaludis	1,772,694	1788	198	95
Methanocorpusculum labreanum	1,804,962	1739	179	91
Methanoculleus marisnigri	2,478,101	2489	205	96
Methanopyrus kandleri	1,694,969	1691	181	98
Methanosaeta thermophila	1,879,471	1696	199	97
Methanosarcina mazei	4,142,816	3347	210	105
Methanosphaera stadtmanae	1,767,403	1534	187	96
Methanospirillum hungatei	3,544,738	3139	203	94
Methanothermobacter thermautotrophicus	1,751,377	1869	186	95
Natronomonas pharaonis	2,749,696	2694	185	87
Picrophilus torridus	1,545,895	1535	159	89
Pyrococcus horikoshii	1,738,505	2061	174	84
Thermococcus kodakarensis	2,088,737	2306	201	94
Thermoplasma volcanium	1,584,804	1526	159	86
Aeropyrum pernix	1,669,696	1700	171	92
Caldivirga maquilingensis	2,077,567	1963	159	79
Hyperthermus butylicus	1,667,163	1602	163	90
Metallosphaera sedula	2,191,517	2256	169	84
Methanosarcina acetivorans	5,751,492	4540	209	106
Pyrobaculum arsenaticum	2,121,076	2298	168	84
Staphylothermus marinus	1,570,485	1570	167	86
Sulfolobus tokodaii	2,694,756	2816	169	83
Thermoproteus neutrophilus	1,769,823	1966	173	89
Nitrosopumilus maritimus	1,645,259	1795	162	83
Nitrososphaera viennensi	2,527,938	3123	172	88
Candidatus Korarchaeum cryptofilum	1,590,757	1602	156	84
Candidatus Caldiarchaeum subterraneum	1,680,938	1742	143	79
Lokiarchaeum sp.	5,143,417	5384	157	87
Prometheoarchaeum syntrophicum	4,427,796	3944	164	79
Archaeon LC1Nh	973,463	1162	126	77
Mancarchaeum acidiphilum	952,257	952	116	65
Nanopusillus acidilobi	605,887	587	96	51
Nanoarchaeum equitans	490,885	536	113	71
Amphimedon queenslandica	166,699,561	9827	127	63
Aspergillus niger	34,006,671	10609	115	57

Branchiostoma floridae	**521,895,125**	**28623**	**107**	**54**
Ciona intestinalis	**115,227,301**	**14096**	**107**	**55**
Cryptococcus neoformans	**19,051,922**	**6475**	**105**	**54**
Daphnia pulex	**189,550,516**	**30611**	**114**	**57**
Drosophila melanogaster	**143,726,002**	**27755**	**108**	**58**
Encephalitozoon intestinalis	**2,216,898**	**1799**	**147**	**54**
Homo sapiens	**3,099,706,404**	**71338**	**124**	**60**
Hydra vulgaris	**852,170,992**	**17002**	**104**	**54**
Pediculus humanus	**110,781,312**	**10775**	**98**	**54**
Saccharomyces cerevisiae	**12,157,105**	**5906**	**110**	**55**
Saccoglossus kowalevskii	**775,840,395**	**22111**	**101**	**54**
Schizosaccharomyces pombe	**12,591,251**	**5133**	**113**	**57**
Strongylocentrotus purpuratus	**921,855,793**	**22709**	**109**	**55**
Arabidopsis thaliana	**119,668,634**	**35176**	**151**	**72**
Micromonas commoda	**21,109,336**	**10140**	**149**	**75**
Ostreococcus tauri	**13,032,761**	**7662**	**137**	**74**
Physcomitrella patens	**472,081,022**	**35936**	**158**	**84**
Phytophthora infestans	**228,543,505**	**17797**	**116**	**58**
Paramecium tetraurelia	**72,094,543**	**39642**	**103**	**59**
Acanthamoeba castellanii	**42,019,824**	**14974**	**105**	**56**
Dictyostelium discoideum	**34,204,973**	**13191**	**108**	**60**
Leishmania major	**32,855,089**	**8265**	**79**	**48**
Giardia intestinalis	**12,078,186**	**7364**	**74**	**48**
Candidate division Kazan bacterium	**602,646**	**591**	**66**	**36**
Candidatus Wolfebacteria bacterium GW2011	**984,447**	**1022**	**52**	**31**
Candidatus Campbellbacteria bacterium	**752,630**	**739**	**53**	**30**
Candidatus woesebacteria bacterium	**819,458**	**909**	**62**	**35**
Candidatus Beckwithbacteria bacterium	**1,049,888**	**1113**	**66**	**35**
Candidate division WWE3 bacterium RAAC2	**878,109**	**871**	**54**	**28**
Berkelbacteria bacterium	**915,059**	**992**	**64**	**32**
Candidatus Saccharibacteria aalborgensis	**1,013,781**	**1,048**	**65**	**33**
Candidatus Saccharibacteria bacterium	**1,005,782**	**1,089**	**69**	**32**
Candidatus Saccharibacteria bacterium GW2011_GWC2_44_17	**1,038,683**	**1,052**	**67**	**32**
Candidatus Saccharibacteria bacterium RAAC3_TM7_1	**845,464**	**717**	**62**	**26**
Candidatus Saccharibacteria bacterium YM_S32_TM7_50_20	**1,450,269**	**1,524**	**64**	**28**
Candidatus Saccharibacteria oral taxon TM7x	**705,138**	**633**	**66**	**32**
Candidatus Minimicrobia massiliensis	**948,334**	**935**	**64**	**31**
TM7 phylum sp. oral taxon 488 AC001	**889,964**	**913**	**66**	**31**
TM7 phylum sp. oral taxon 488 AC002	**847,999**	**851**	**67**	**32**
TM7 phylum sp. oral taxon 488 CM006	**893,660**	**915**	**66**	**32**
TM7 phylum sp. oral taxon 952	**705,178**	**776**	**66**	**31**
TM7 phylum sp. oral taxon 955	**842,202**	**916**	**66**	**33**
TM7 phylum sp. oral taxon 957	**782,344**	**711**	**67**	**32**
Candidate division SR1 bacterium Aalborg AAW-1	**1,044,756**	**993**	**54**	**31**
Candidate division SR1 bacterium RAAC1	**1,177,760**	**1059**	**60**	**36**
Candidatus Gracilibacteria bacterium HOT-871	**1,184,529**	**1112**	**66**	**37**

Candidatus gracilibacteria bacterium	1,343,103	1,196	70	36
Candidatus uzinura diaspidicola	263,431	227	39	29
Candidatus Sulcia muelleri	285,352	251	34	25
Blattabacterium cuenoti	632,490	584	60	37
Candidatus Ishikawaella capsulata	754,729	614	79	36
Blochmannia endosymbiont of Camponotus	773,940	584	69	41
Wigglesworthia glossinidia	719,535	635	68	39
Candidatus Arsenophonus lipoptenae	836,724	625	73	38
Candidatus Moranella endobia	538,294	406	70	43
Buchnera aphidicola	655,725	564	69	38
Candidatus Baumannia cicadellinicola	759,425	689	79	40
Candidatus Profftella armatura	464,857	366	60	38
Candidatus Portiera aleyrodidarum	359,359	267	36	27
Candidatus Carsonella ruddii	174,017	192	8	7
Candidatus Hodgkinia cicadicola	150,297	170	4	4
Candidatus Tremblaya princeps	138,931	116	15	12
Candidatus Zinderia insecticola	208,564	206	15	12
Rickettsia conorii	1,260,331	1,136	80	42
Rickettsia fournieri	1,447,740	1,282	91	45
Rickettsia tamurae	1,453,216	1,295	91	45
Neorickettsia helminthoeca	884,232	868	75	39
Candidatus Nasuia deltocephalinicola	144,602	159	7	4
Tropheryma whipplei	927,303	808	82	39
Parachlamydia acanthamoebae	3,072,383	2788	95	45
Chlamydia pneumonia	1,230,230	1052	73	38
Candidatus Babela massiliensis (TM6)	1,118,422	981	66	35
Acidobacterium capsulatum	4,127,356	3382	120	45
Granulicella mallensis	6,237,577	4815	123	44
Hydrogenobaculum sp.	1,516,013	1629	90	42
Aquifex aeolicus	1,590,791	1522	104	42
Lactobacillus kefiranofaciens	2,173,634	2026	111	47
Lactobacillus delbrueckii	1,864,998	2096	107	43
Lactobacillus timonensis	1,678,325	1,527	110	45
Acidaminococcus intestini	2,487,765	2404	108	43
Thermoanaerobacter tengcongensis	2,689,445	2588	118	41
Exiguobacterium sibiricum	3,040,786	3007	135	46
Clostridium massiliodielmoense	2,759,335	2491	122	48
Clostridium botulinum	3,858,511	3669	124	46
Clostridium acetobutylicum	3,903,260	3652	121	43
Bacillus cereus	5,419,036	5398	139	52
Bacillus subtilis	4,215,606	4331	135	49
Bacillus tuaregi	4,863,531	4440	136	50
Bacillus mediterranensis	3,340,964	3219	134	48
Rhodopirellula baltica	7,145,576	7325	106	46
Thermus thermophilus	2,116,056	1973	105	48
Deinococcus perariditilitoris	4,513,714	3636	122	49

Acidothermus cellulolyticus	**2,443,540**	**2157**	**107**	**45**
Mycobacterium abscessus	**5,090,491**	**4920**	**116**	**47**
Salinispora arenicola	**5,786,361**	**4917**	**126**	**52**
Streptomyces coelicolor	**9,054,847**	**7825**	**128**	**46**
Streptomyces bingchenggensis	**11,936,683**	**10022**	**136**	**46**
Chlorobium tepidum	**2,154,946**	**2255**	**105**	**44**
Chlorobium phaeobacteroides	**3,133,902**	**2650**	**115**	**44**
Gramella forsetii	**3,798,465**	**3584**	**97**	**43**
Bacteroides thetaiotaomicro	**6,293,399**	**4712**	**100**	**44**
Bacteroides congonensis	**6,373,337**	**4723**	**103**	**43**
Chitinophaga pinensis	**9,127,347**	**7192**	**118**	**47**
Opitutus terrae	**5,957,605**	**4612**	**100**	**40**
Thermosipho melanesiensis	**1,915,238**	**1879**	**108**	**46**
Chloroflexus aurantiacus	**5,258,541**	**3853**	**112**	**41**
Caldilinea aerophila	**5,144,873**	**4,119**	**121**	**45**
Roseiflexus castenholzii	**5,723,298**	**4330**	**115**	**40**
Gloeobacter violaceus	**4,659,019**	**4430**	**116**	**45**
Trichormus variabilis	**7,105,752**	**5039**	**114**	**40**
Nostoc punctiforme	**9,059,191**	**6087**	**119**	**45**
Sulfurimonas denitrificans	**2,201,561**	**2096**	**94**	**41**
Arcobacter butzleri	**2,341,251**	**2259**	**92**	**44**
Gallibacterium anatis	**2,694,139**	**2494**	**133**	**50**
Helicobacter acinonychis	**1,557,588**	**1612**	**95**	**42**
Arcobacter nitrofigilis	**3,192,235**	**3126**	**103**	**42**
Desulfovibrio vulgaris	**3,773,159**	**3379**	**127**	**53**
Gluconacetobacter diazotrophicus	**3,914,947**	**3472**	**143**	**46**
Caulobacter crescentus	**4,042,929**	**3886**	**141**	**49**
Rhodospirillum rubrum	**4,406,557**	**3791**	**138**	**47**
Dechloromonas aromatica	**4,501,104**	**4171**	**128**	**51**
Shewanella denitrificans	**4,545,906**	**3754**	**132**	**49**
Psychromonas ingrahamii	**4,559,598**	**3545**	**136**	**50**
Rhodobacter sphaeroides	**4,602,977**	**3885**	**142**	**47**
Geobacter bemidjiensis	**4,615,150**	**4051**	**127**	**52**
Aeromonas hydrophila	**4,744,448**	**4122**	**140**	**49**
Chromobacterium violaceum	**4,751,080**	**4407**	**136**	**53**
Bordetella petri	**5,264,383**	**5,035**	**135**	**46**
Klepsiella aerogenes	**5,280,350**	**4912**	**161**	**51**
Xanthomonas axonopodis	**5,296,525**	**4245**	**128**	**50**
Polaromonas naphthalenivorans	**5,366,143**	**4084**	**126**	**52**
Colwellia psychrerythraea	**5,373,180**	**4910**	**139**	**50**
Pseudomonas syringae	**6,093,698**	**5482**	**141**	**50**
Pseudomonas aeruginosa	**6,264,404**	**5571**	**143**	**53**
Achromobacter xylosoxidans	**6,813,182**	**6459**	**139**	**49**
Ralstonia eutropha/Cupriavidus necator	**8,480,857**	**6065**	**149**	**49**
Burkholderia gladioli	**8,899,459**	**6704**	**149**	**53**
Microvirga massiliensis	**9,172,134**	**7977**	**150**	**47**
Bradyrhizobium diazoefficiens	**9,105,828**	**8,317**	**150**	**48**

Sorangium cellulosum	13,033,779	9384	138	54
spirochaeta smaragdinae	4,653,970	4219	124	56
Treponema pallidum DAL-1	1,139,971	1,059	76	39
Treponema phagedenis	3,372,116	2,930	113	52
Treponema denticola	2,795,521	2626	106	47
Fusobacterium nucleatum	2,174,500	2067	102	45
Spodoptera_frugiperda	156,922	123	1	0
AfricanSwineFeverVirus	182,284	156	0	0
Ambystoma tigrinum	106,332	95	0	0
Aedes Invertebrate iridescent virus	191,100	126	0	0
Marseillevirus marseillevirus T19	368,454	457	2	0
Bodo saltans virus	1,385,869	1143	7	3
Crovirus	617,453	544	5	2
Acanthamoeba polyphaga mimivirus	1,181,549	979	8	2
Tupanvirus soda lake	1,439,508	1359	14	7
Yasminevirus	2,126,343	1624	10	5
ATCV1	288,047	860	1	0
Ectocarpus siliculosus	335,593	240	0	0
Orpheovirus	1,473,573	2398	3	2
Cedratvirus massiliensis	589,068	574	2	0
Pithovirus sibericum ORFome	610,033	467	1	0
Amsacta moorei entomopoxvirus	232,392	294	1	0
Faustovirus E12	466,265	492	2	0
Medusavirus	362,811	461	0	0
Pandoravirus salinus	2,473,870	2542	3	1
Mollivirus sibericum	651,523	523	2	0
Kaumoebavirus	350,731	264	1	0
Pacmanvirus	395,405	465	2	0
Homo sapiens	16,569	13	0	0
Reclinomonas americana	69,034	67	8	7
Saccharomyces cerevisiae	89,507	18	0	0
Anopheles gambiae	15,363	13	0	0
Acanthamoeba castellanii	41,591	40	1	1
Pediculus humanus	40,023	11	0	0
Ostreococcus tauri	44,237	43	0	0
Ostreococcus tauri	71,666	60	0	0
Micromonas commoda	72,585	57	0	0
